# The Mechanisms of the Initiation Step in Ring-Opening Polymerization of β-Lactones: A Review

**DOI:** 10.3390/polym18121488

**Published:** 2026-06-13

**Authors:** Zbigniew Grobelny, Sylwia Golba, Justyna Jurek-Suliga

**Affiliations:** 1 Institute of Chemistry, University of Silesia, 40-007 Katowice, Poland; zbigniew.grobelny@us.edu.pl; 2 Institute of Materials Science, University of Silesia, 40-007 Katowice, Poland; sylwia.golba@us.edu.pl

**Keywords:** β-lactones, ring-opening polymerization (ROP), mechanism of initiation, coordination–insertion mechanism

## Abstract

Ring-opening polymerization (ROP) of β-lactones yields biodegradable and bioresorbable polyesters exhibiting potential utility in medicine and environmental protection. β-butyrolactone (BL) is especially interesting, as it yields polymers analogous to natural poly(3-hydroxybutyrates) produced by bacteria, fungi, and enzymes in nature. The biopolymer produced by these microorganisms is isotactic. While it can be synthesized biotechnologically through the bacterial fermentation of substrates, such as sucrose, corn, and sugar, laboratory production typically involves the ring-opening polymerization of BL. However, the latter process is mainly atactic, syndiotactic, or partly isotactic, and other β-substituted β-lactones are less well-known. Ring-opening polymerization is an excellent pathway for the production of poly(β-lactones). This critical review presents the different conditions required to synthesize poly(β-lactones) and a broad overview of the different kinds of ROPs, i.e., anionic, cationic, coordinative, supramolecular-based, and enzymatic processes. A great variety of initiators/catalysts have been studied, covering both metal-based and metal-free systems (organo- and biocatalysts). In this review, several mechanisms of β-lactone polymerization are presented and discussed, especially with regard to the processes’ initiation steps.

## 1. Introduction

Among all cyclic esters, β-propiolactone (PL) is the simplest lactone and has the smallest four-membered ring. This lactone and its β-substituted derivatives, especially β-butyrolactone (BL), are of great interest because they play a role in ring-opening polymerization (ROP), which results in linear biocompatible and biodegradable aliphatic polyesters [1]. These compounds are suitable for many environmental and biomedical applications, such as fine chemicals, biofuels, bioplastics, implant bio-materials, and nanocarriers for drug delivery [2,3,4].

Poly(3-hydroxybutyrate) (PHB), the most common biological poly(β-hydroxyalcanoate) (PHA), is naturally produced by many microorganisms. It is a perfectly isotactic (comprising a structure with only (*R*)-configuration), highly crystalline thermoplastic polymer (T_m_ = 180 °C), and is generally synthesized through biotechnological processes. However, it has low thermostability, which limits its potential industrial utility [5].

**Scheme 1:**
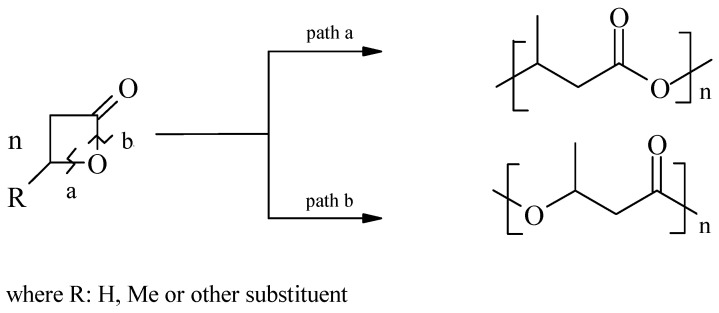
Monomer ring-opening during propagation in β-lactone polymerization [6].

In general, β-lactones can be prepared using ROP methods, in which propagation occurs via monomer ring-opening in two positions—i.e., O-alkyl (path a) or O-acyl (path b)—as shown in Scheme 1 [6].

Several different mechanisms have been proposed for the propagation step, which depend on the initiator used.

1. Anionic ROP: active chain-end (carboxylate anion) mechanism (path a).
2. Cationic ROP:
  (a) Active chain-end (cyclic tertiary oxonium cation) (ACE) mechanism (path a).
  (b) Activated monomer (AM) mechanism (path b).
3. Coordinative ROP: coordination–insertion mechanism (path b).
4. Supramolecular-based ROP: non-covalent activated monomer/initiator mechanism (path b).
5. Enzymatic ROP: activated monomer mechanism (path b).

Anionic, cationic, supramolecular, and enzymatic polymerization of rac-BL results in atactic polyesters exclusively. The most convenient method for the synthesis of natural isotactic PHB analogs is the ROP of enantiomerically pure β-substituted-β-lactones, i.e., *(R)*- or *(S)*-β-BL [7]. However, they are much more expensive than *rac*-β-substituted-β-lactones. Using certain initiators and under specific conditions, it has been possible to achieve some level of stereocontrol during the coordinative ROP of racemic monomers and synthesize products with variable *iso*- or *syndio*-tacticities. However, the resulting synthetic polymers differ strongly from the purely isotactic materials obtained biotechnologically.

Many different initiators and catalyst/initiator systems, including metal-based, metal-free, or enzymatic systems, have been used for the ROP of β-lactones [1,2,3,4,5,6,7], as they have been of interest to scientists investigating the polymerization of cyclic esters. This article presents a broad overview of the mechanisms of the initiation steps employed when determining the course of the ring-opening polymerization of β-lactones.

## 2. Anionic ROP

### 2.1. Initiation Using Ionic Alkali Metal Salts

#### 2.1.1. Strong Bases

Dale [8] studied PL polymerization initiated with *t*-BuOK and, unexpectedly, did not find any *t*-butoxy starting groups derived from the initiator; instead, unsaturated groups were observed in the prepared polymers. Kricheldorf et al. [9] investigated bulk BL polymerization in the presence of *t*-BuOK at 50 °C or in deuterated solvents such as CDCl_3_, C_6_D_5_NO_2_, and DMSO-*d*_6_. They observed that deprotonation of the monomer is favored in highly polar solvents and at higher temperatures. It was proposed that high yields of *trans*-crotonate starting groups result from direct deprotonation of the monomer by the initiator. Crotonate anions can react as anionic initiators via nucleophilic attack on C3 of the monomer.

**Scheme 2:**
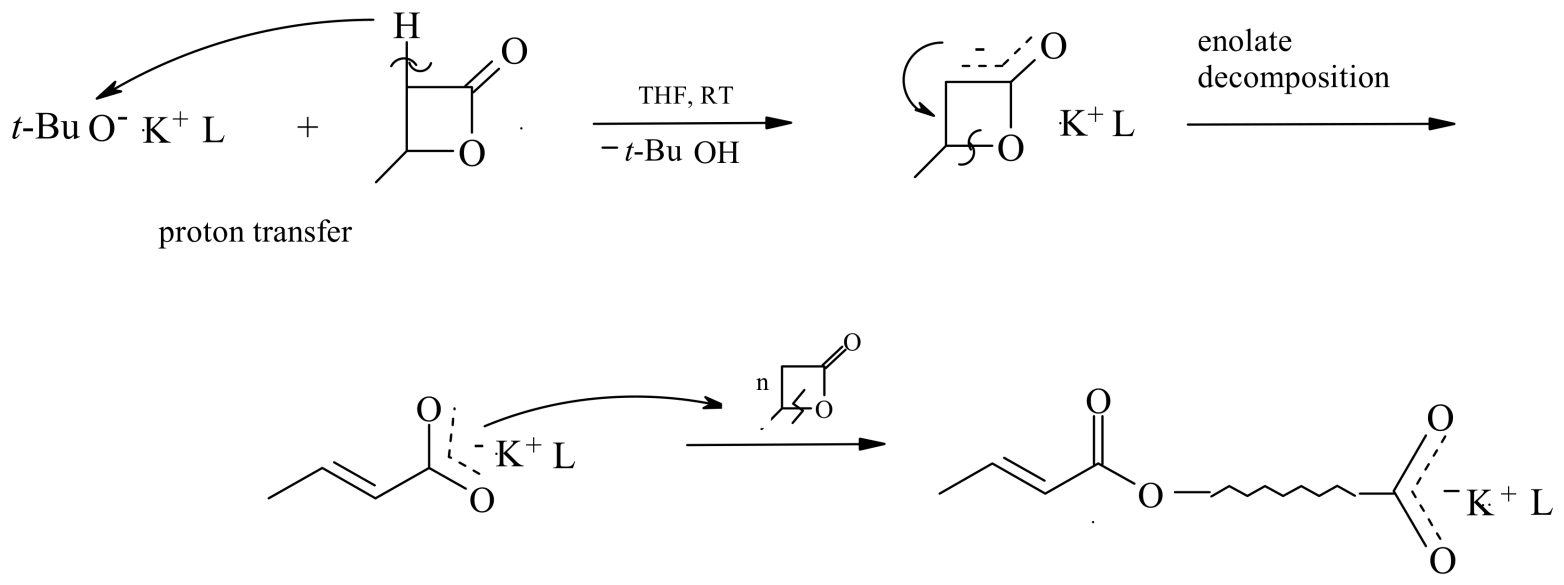
Initiation of BL polymerization with solubilized potassium *t*-butoxide activated by 18C6 (L) [11].

Jedliński et al. [10] reported that in the presence of *t*-BuOK activated by a strong cation complexing agent—i.e., coronand 18-crown-6 (18C6)—it was possible to realize the polymerization of BL in THF. It could be realized at room temperature due to the formation of highly reactive crown-separated ion pairs. However, the authors proposed quite a different initiation course. The formation of two kinds of macromolecules was postulated—i.e., those possessing 3-hydroxybutyrate and crotonate starting groups—where the latter formed, unexpectedly, in reactions other than monomer deprotonation. Therefore, we repeated this experiment under the same conditions and observed that initiation proceeded mainly through deprotonation of the monomer by the initiator (Scheme 2) [11].

Only a small number (~4%) of macromolecules possessed 3-hydroxybutyrate starting groups. This phenomenon is explained in Section 2.1.2. Moreover, macromolecules with crotonate starting groups and -COOH end groups were not detected using ^13^C NMR and MALDI-TOF techniques after quenching with methyl iodide. This indicated that in the studied polymerization side reactions—i.e., chain transfer to *t*-BuOH (as the reaction product)—the monomer and polymer are strongly limited. This results from the low basicity of carboxylate end groups and their ability to form complexes with *t*-BuOH. The polymer prepared at [M]_0_[I]_0_ = 20 had molar mass M_n_(GPC) = 1750 (M_caled_= 1722), polymer dispersity M_w_/M_n_ = 1.16, and initiator efficiency M_caled_/M_n_ equal to 0.99. The yield of polymerization was 99.5% after 25 h.

**Scheme 3:**
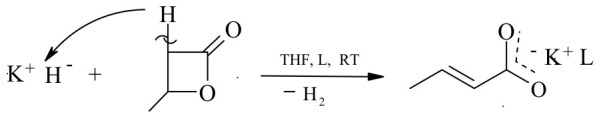
Initiation of BL polymerization with suspension of potassium hydride activated by 18C6 (L). The formation of potassium enolate was omitted [12].

However, exclusive deprotonation of PL and BL occurred due to the use of KH activated by 18C6 (Scheme 3). In this case, K^+^H^−^ reacts quantitatively with the monomer and gaseous hydrogen evolves as the reaction product [12].

Potassium hydride, in contrast to potassium *t*-butoxide, appeared to be completely insoluble in THF, even in the presence of a strong complexing ligand such as 18C6. Polymerization initiates on the surface of the powdered KH upon contact with the solvent, and propagation then continues in the solution. Polymerizations were performed in THF solution or in bulk. Equimolar amounts of hydrogen evolved during polymerization. The synthesized polymers exhibited M_n_ = 2900–15,900, determined using the VPO method (almost the same as that calculated), and M_w_/M_n_ = 1.13–1.06. The yields after 48–96 h were 93–97%, and similar results were obtained through the polymerization of PL.

The authors of [12] suggested that previously described polymerizations are “living” processes and lead to new polymer macromonomers with unsaturated and carboxylic end groups after quenching with methanol.

We have observed similar phenomena in BL polymerization initiated under the same conditions as other systems, i.e., NaH/C222, KH/C222, Ph_2_PK/18C6, Ph_3_HBK/18C6, and (Me_3_Si)_2_ NK/18C6 (where C222 denotes the cryptand). However, species such as NaH, NaH/15C5, NaH/18C6, KH, KH/15C5, and Ph_4_BK/18C6 did not initiate polymerization [11].

**Scheme 4:**
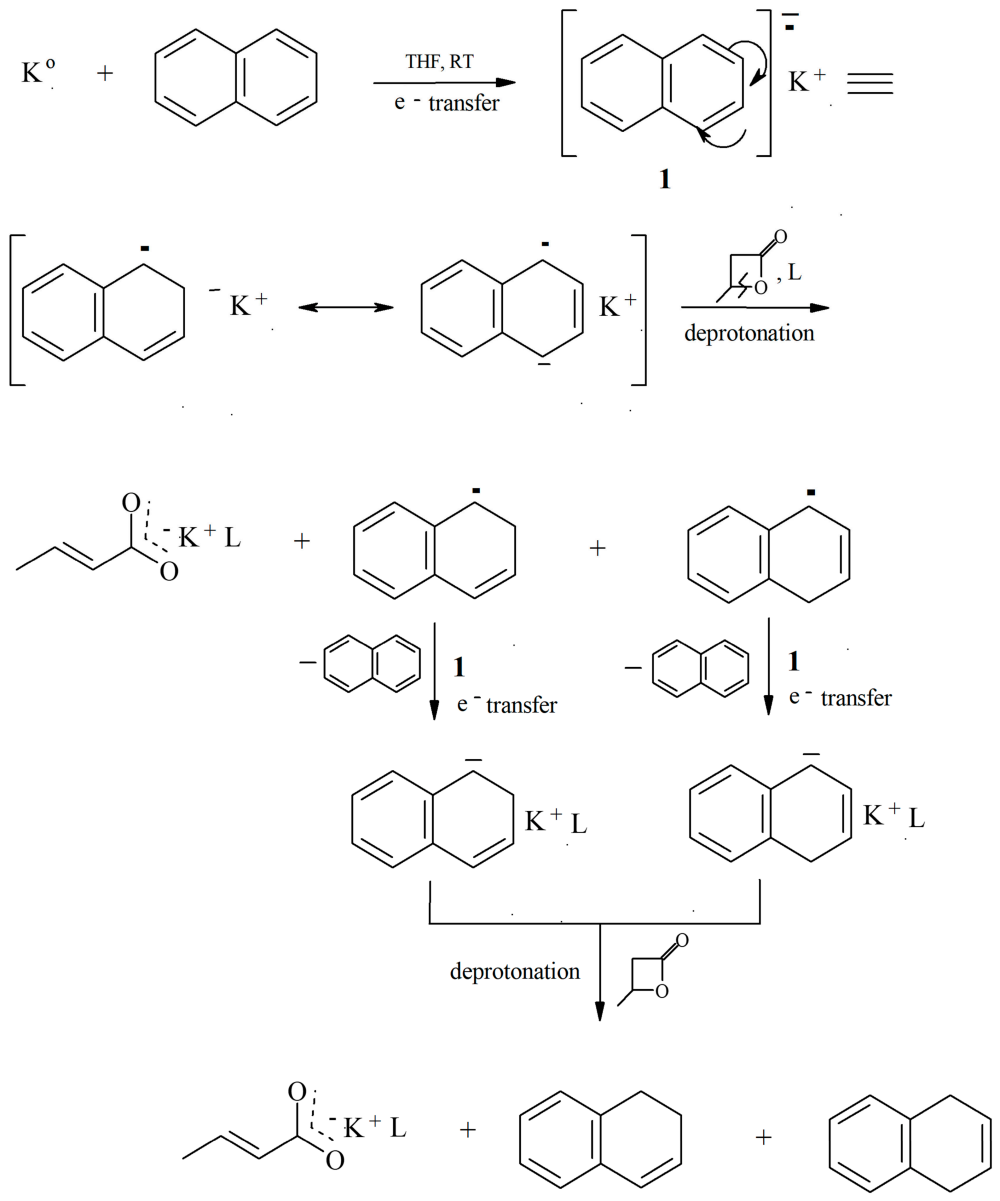
Initiation of BL polymerization with potassium naphthalenide activated by 18C6 (L) [13].

An interesting example of “living” anionic polymerization of BL is the process mediated by potassium naphthalenide in the presence of 18C6 or C222. The mechanism of polymerization in this case has been elucidated by Jedliński et al. [13]. Note that, in this case, a complexing agent was added to the initiator after its preparation in order to avoid the rapid formation of potassium anions (K^−^) in the reaction of metallic potassium with the ligand. The course of the process is presented in Scheme 4. Organopotassium compounds formed in this system as intermediates deprotonate the monomer to produce potassium crotonate, which acts as the actual polymerization initiator. Thus, it was indicated that naphthalene radical anions did not react with the monomer as an electron transfer reagent. Authors have also stated [13] that the synthesis of “living” poly(β-hydroxybutyrate) macromonomers with unsaturated end groups via anionic polymerization can be achieved through the use of potassium naphthalenide.

A small number of crotonate groups can originate from chain transfer to the monomer, i.e., through the deprotonation of the monomer by carboxylate end groups on the polymer chains [14]. The second source of crotonization is intermolecular degradation of the polymer, which proceeds through the abstraction of a proton at the C2 carbon of the polymer chain by the carboxylate end group (polymer deprotonation) [15,16]. Both reactions are undesired because they lead to a decrease in the molecular mass of the polymers. However, crotonization caused by these side reactions is expected to be rather low due to the low basicity of carboxylate anions.

In the studied systems containing potassium naphthaleneide, methanol was added as a quenching agent. The end-group analysis revealed the presence of one carboxylic end group and one crotonate starting group in each polymer chain. The polymers obtained at [M]_0_/[I]_0_ = 40 had molar mass M_n_(VPO) = 2800 (M_caled_ = 3444) and dispersity M_n_/M_w_ = 1.28. The yield of polymerization was 90% after 96 h [12,13].

#### 2.1.2. Nucleophilic Reagents

**Scheme 5:**
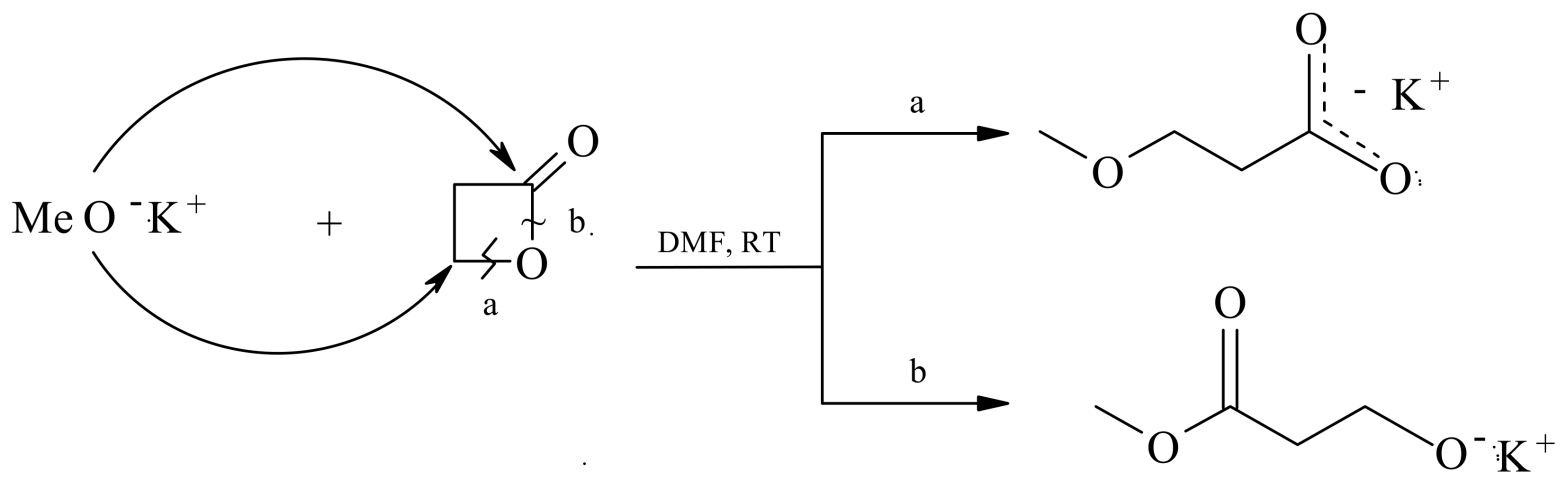
Initiation of PL polymerization with potassium methoxide [17].

Penczek et al. studied initiation with nucleophilic reagents [17]. They carried out PL polymerization in the presence of a strong nucleophile, i.e., MeOK, in DMF solution at room temperature. The signals of the starting groups in the ^1^H NMR spectrum indicated that two modes of monomer ring-opening participate in the initiation process: O-alkyl and O-acyl bond scission, which lead to carboxylate- and alkoxide-type active centers, respectively (Scheme 5).

**Scheme 6:**
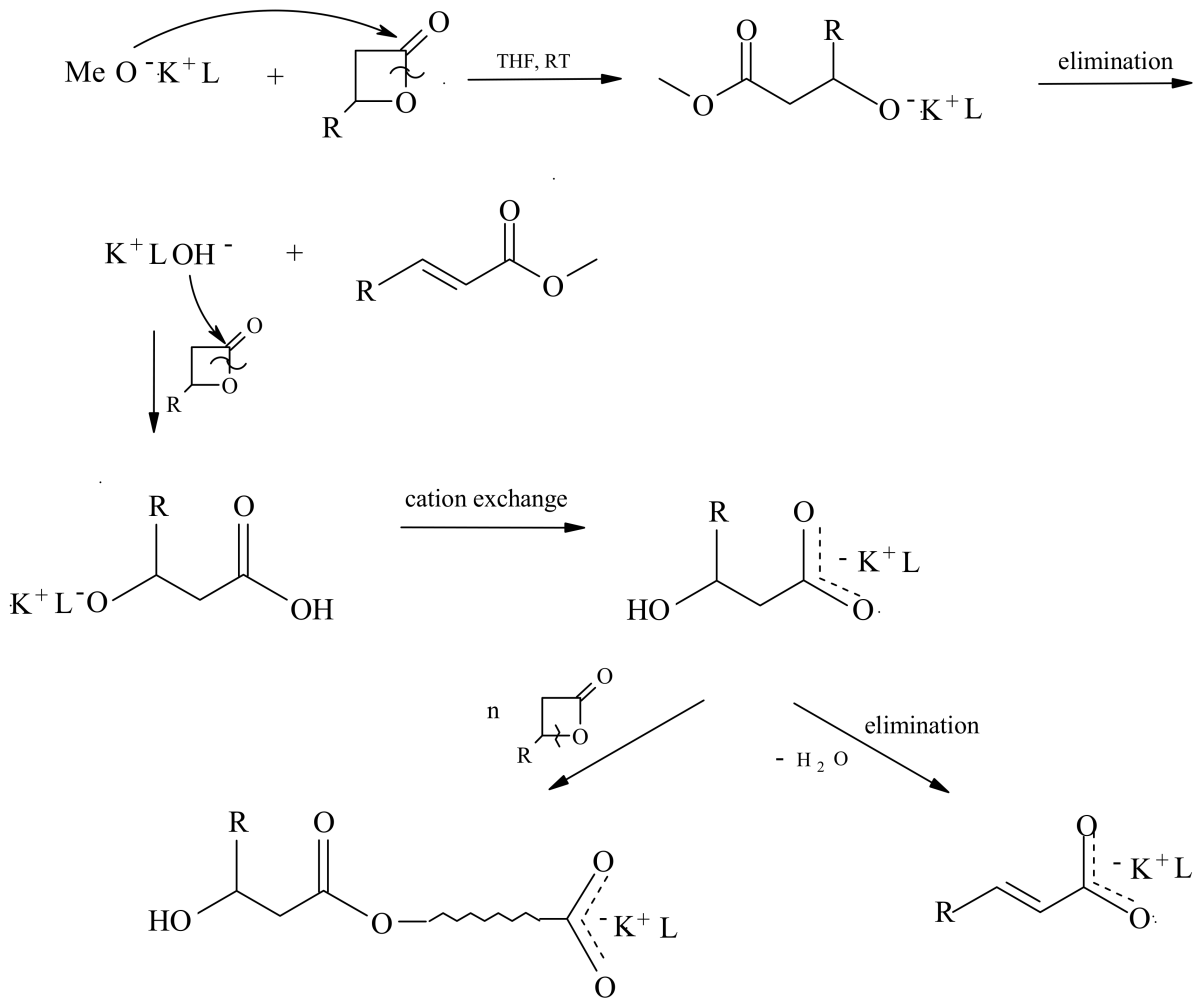
Initiation of PL and BL polymerization with potassium methoxide activated by 18C6 (L) [19]. R is H or Me.

Under the conditions applied in this work, these species formed in almost equal proportions. In this manner, at the propagation step, the alkoxide anions were completely converted into carboxylate anions (<0.1% of alkoxide anions were left after 10 steps of propagation). However, the authors did not explain this mechanism. Propagation proceeds via O-alkyl bond scission through an attack on the CH_2_ group adjacent to the oxygen atom. The same result was obtained in the presence of DB18C6 as a cation complexing agent. Data concerning molecular mass, disparity, initiator efficiency, and yield were not presented by the authors. Later, Penczek et al. [18] initiated the polymerization of PL with MeOH in CH_2_Cl_2_ at RT. ^1^H NMR analysis revealed the presence of four kinds of starting groups in macromolecules, i.e., CH_3_OCH_2_CH_2_^−^, CH_3_OC(O)^−^, HOCH_2_CH_2_^−^, and CH_2_=CHCOO^−^. Approximately 10% of the macromolecules contained starting groups formed due to the incorporation of the initiator. Thus, the proportion of the two reaction paths appears to be solvent-dependent. In much less polar CH_2_Cl_2_, alkoxide anions also participate in side reactions (proton transfer or the elimination of hydroxyl anions from alkoxides). However, by further changing the reaction conditions (i.e., the solvent and ligand), quite a different initiation course with MeOK was observed by Jedliński et al. [19] for PL and BL (Scheme 6). Polymerization was carried out in THF in the presence of 18C6, resulting in polymers with M_n_ = 4000 and M_w_/M_n_ = 1.20 and high yield (~95%) after 28 h.

The authors proposed that in the first step of the process, MeOK mediates O-acyl bond cleavage, followed by the elimination of KOH and formation of methyl crotonate in the case of BL. Then, the former reacts with the next monomer molecule via O-acyl bond cleavage. The reaction product then transforms into the potassium salt of a hydroxycarboxylic acid, which initiates further polymerization. In the second reaction, H_2_O is eliminated, resulting in potassium *trans*-crotonate, which becomes the second initiator of polymerization. A similar process was proposed by Jedliński et al. [10,20] for PL and BL polymerization with *t*-BuOK activated by 18C6. However, our recent studies did not confirm the latter [11]: BL polymerization was initiated in this case mainly through monomer deprotonation, but the elimination of KOH was negligible.

**Scheme 7:**
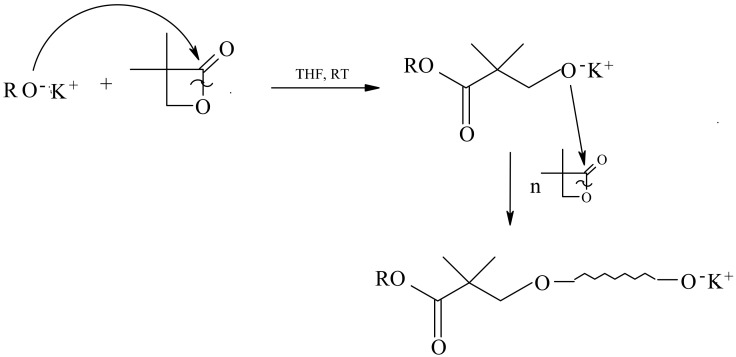
Initiation of pivalolactone polymerization with potassium alkoxides [21]. R is Me or *t*-Bu.

PL and BL polymerization proceeds via carboxylate active centers, independent of initiation course. However, in the case of some other β-lactones (i.e., α, α-disubstituted β-lactones), the mechanism is different. For example, in the polymerization of pivalolactone initiated with MeOK or *t*-BuOK, the nucleophilic attack of an initiator occurs on the carbonyl atom of the monomer, resulting in O-acyl bond cleavage with the formation of alkoxide anions as propagating species [21] (Scheme 7). In this case, the elimination reaction does not occur due to the absence of hydrogen at the C-2 monomer molecule.

Propagation proceeds according to a living mechanism, which allows for control of the molar masses of the prepared polymers.

**Scheme 8:**
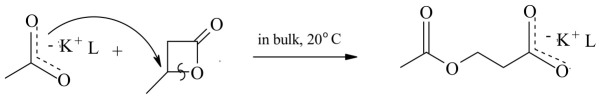
Initiation of BL polymerization with potassium acetate activated by 18C6 (L) [13].

Different results were obtained in the polymerization of BL initiated with potassium acetate, which is a weak nucleophile (Scheme 8) [13]. In this system, initiation proceeds exclusively via O-alkyl bond scission.

The ^1^H NMR spectrum revealed the absence of crotonate group signals. The polymer obtained at [M]_0_/[I]_0_ = 40 with 92% yield after 100 h had M_n_(GPC) = 2800 and dispersity equal to 1.29. Moreover, in the polymerization of BL with potassium acetate activated by DB18C6 in THF at RT, the crotonate groups were determined to originate from chain transfer to the monomer. The author of [22] suggested that this is a convenient method for the controlled synthesis of polymers with M_n_ up to 20,000 and M_w_/M_n_ = 1.06 − 1.20 (>95% yield after 54 h). Moreover, other simple sodium and potassium carboxylate salts activated by coronands can also be used to initiate the “living” polymerization of PL, realizing quantitative initiation [22].

A similar phenomenon was observed when monopotassium salts of dicarboxylic acids—namely, malonic and diglycolic ones—were used to initiate BL polymerization in THF solution [11]. Both polymers were prepared at [M]_0_/[I]_0_ = 20, with high yield (99%) and very low unsaturation levels (~2 mol%) and dispersity (~1.17). The M_n_(SEC) values of the polymers were 2600 and 1800, respectively. This effect was presumably due to the difference in efficiency between the initiators (0.65 and 0.95, respectively). The yields were ~95% after 40 h.

All polymers synthesized under anionic processes at RT are amorphous and atactic. Considering this, in 1996, Jedliński et al. [23] reported that polyesters produced through the anionic polymerization of racemic BL ((*R,S*)−β−butyrolactone) at −10 °C using the aforementioned potassium methoxide or acetate salts activated by 18C6 are predominantly syndiotactic. A similar effect was obtained in the presence of tartarate diesters as a polar addition at 20 °C. Moreover, *(R)*-β-butyrolactone polymerizes with inversion of the configuration at 20 °C and without additives, yielding highly crystalline isotactic poly*(S)*-β-butyrolactone.

#### 2.1.3. Nucleophilic Bases

**Scheme 9:**
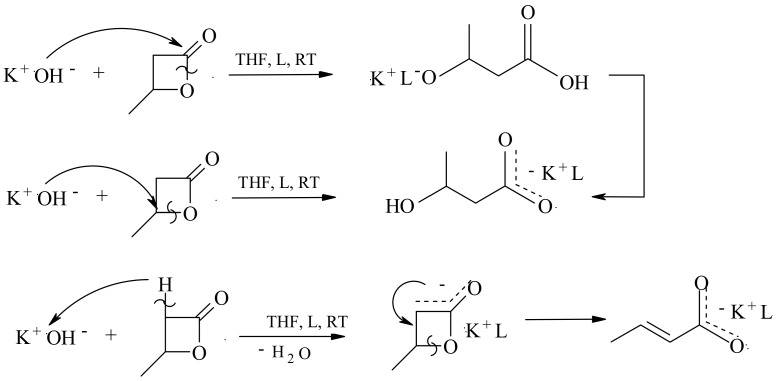
Three possible pathways for the initiation of BL polymerization mediated through the suspension of potassium hydroxide activated by 18C6 (L) [24].

An excellent example of this type of initiation is BL polymerization initiated with anhydrous-powdered KOH activated by 18C6 [24]. Using MALDI–TOF mass spectrometry, we confirmed the formation of two kinds of macromolecules in this process. They contain hydroxyl and *trans*-crotonate starting groups. Thus, we proposed that KOH reacts with BL by O-acyl bond cleavage, resulting in an alkoxide with a carbonyl group that transforms into a carboxylate with a hydroxyl group. The latter may also form directly via O-alkyl bond cleavage (Scheme 9). In these reactions, KOH behaves as a nucleophilic reagent but can also react with the monomer as a base through its deprotonation. The polymer exhibited a molar mass of M_n_ = 2000, dispersity of M_w_/M_n_ = 1.23, and initiator efficiency of M_calc/_M_n_ = 0.86 (98% yield after 25 h).

**Scheme 10:**
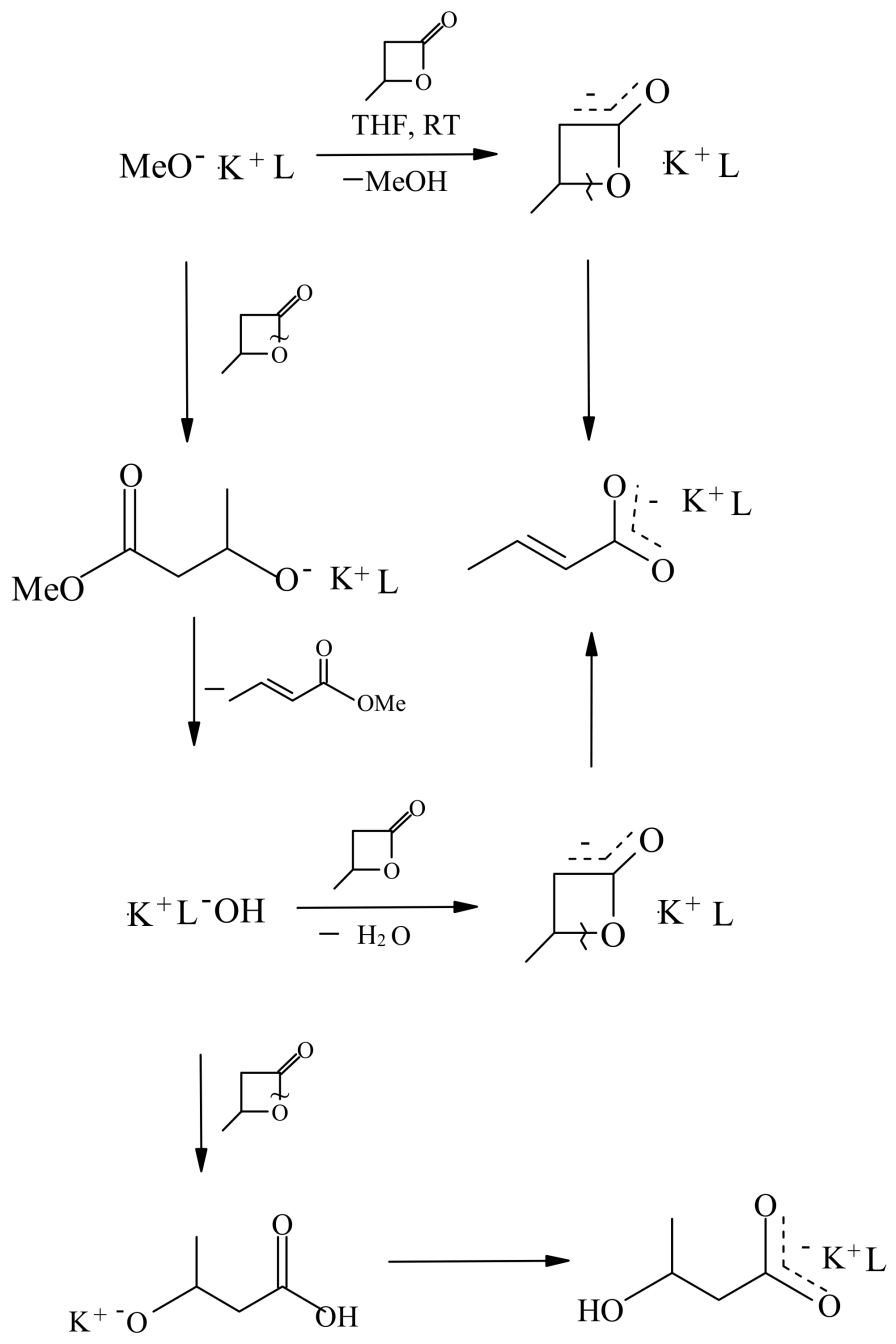
Initiation of BL polymerization with potassium methoxide activated by 18C6 (L) [11].

KOH was postulated earlier, by Jedliński et al. [19], as an intermediate formed during BL polymerization mediated by MeOK. Recently [11], we repeated this process and observed the formation of the same kinds of macromolecules as in the system containing KOH. Based on the results obtained, we rationalized the course of the process (Scheme 10) [11]. It was proposed that MeOK behaves in the initiation step as KOH, i.e., it reacts as a nucleophilic base. The yield of polymerization was 99% after 35 h, and the polymer exhibited a molar mass of M_n_ = 1800, dispersity of M_w_/M_n_ = 1.21, and initiator efficiency of M_calc/_M_n_ = 0.91. Similar results were obtained when using EtOK or *i*-PrOK as initiators [11]. Macromolecules with alkoxy starting groups were not detected in the studied processes, which indicates that the potassium alkoxides used did not participate in propagation.

**Scheme 11:**
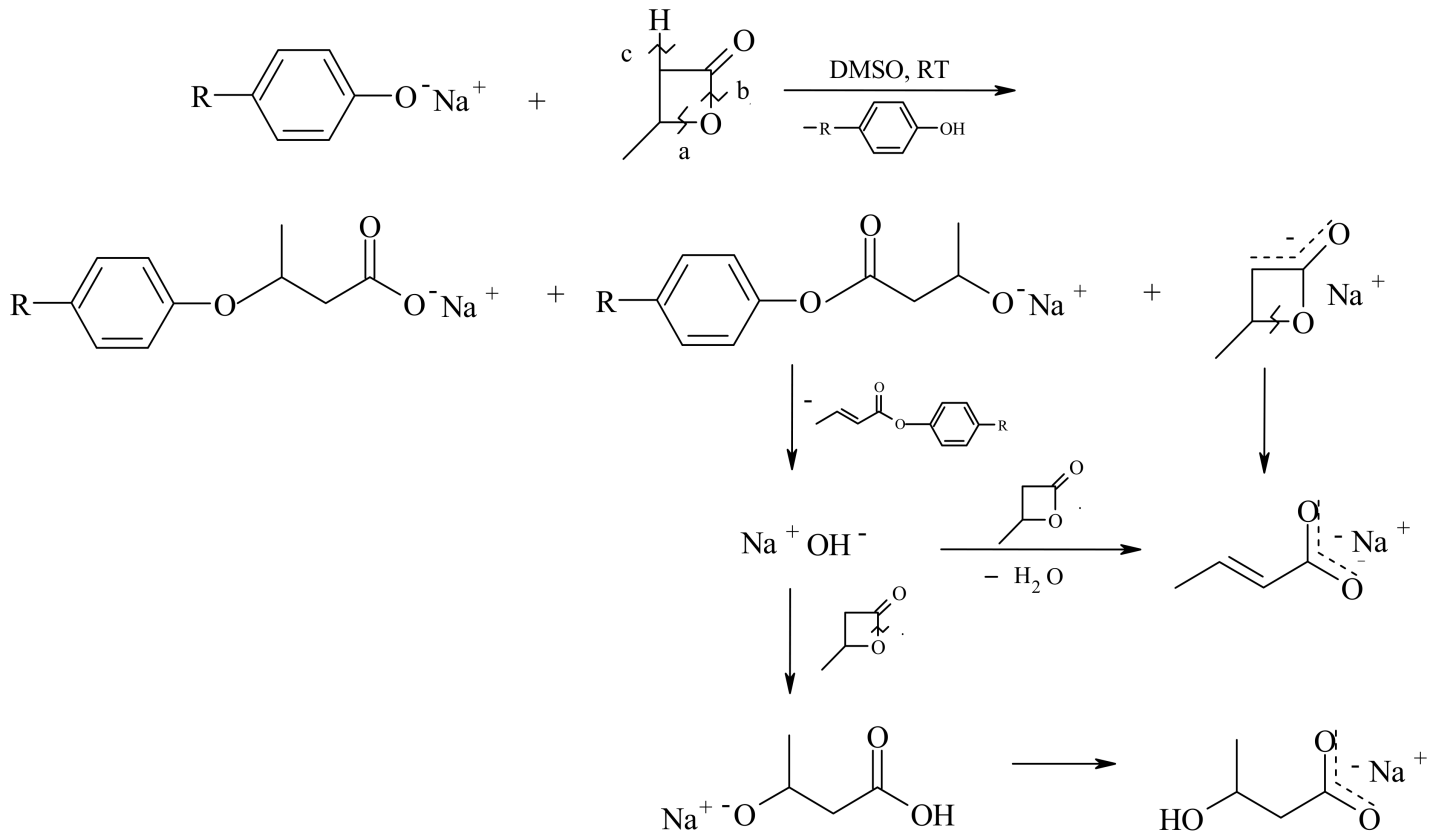
The initiation of BL polymerization with sodium phenoxides occurs in three ways [25]. R is H, NO_2_, Cl, or MeO.

Recently, it has been reported [25] that sodium phenoxide and its selected derivatives with different basicity and nucleophilicity, such as sodium *p*-chlorophenoxide, *p*-methoxyphenoxide, and *p*-nitrophenoxide, are effective initiators of BL polymerization under mild conditions, i.e., in DMSO solution at RT. Phenoxides as initiators were found to behave as weak or strong nucleophiles, as well as bases. All resulting polyesters possessed three kinds of starting groups, i.e., phenoxy, hydroxy, or crotonate groups. They were formed via O-alkyl or O-acyl bond cleavage, followed by the elimination of NaOH and monomer deprotonation, respectively (Scheme 11).

The main factor determining the mechanism of initiation is the basicity/nucleophilicity ratio of the phenoxides used (*p*-NO_2_PhO^−^ < *p*-ClPhO^−^ < PhO^−^ < *p*-MeOPhO^−^). It was observed that sodium *p*-nitrophenoxide, with the lowest basicity, reacts mainly as a weak nucleophile, yielding macromolecules with phenoxy starting groups. However, the use of sodium *p*-methoxyphenoxide, the most basic but weak nucleophilic initiator, generates macromolecules predominantly composed of hydroxyl starting groups.

**Scheme 12:**
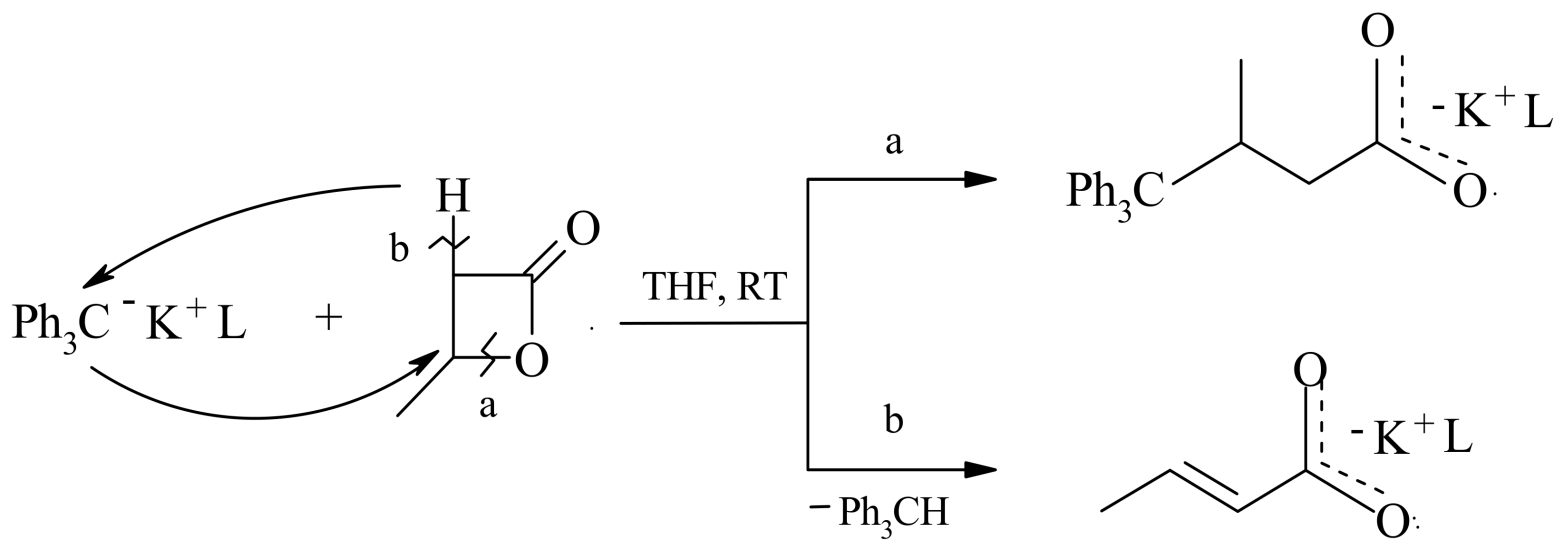
The initiation of BL polymerization with triphenylmethylpotassium activated by 18C6 (L) occurs in two ways [11].

Another example of a nucleophilic base initiating BL polymerization is Ph_3_CK, which opens the monomer ring in the O-alkyl position and deprotonates the monomer (Scheme 12) [11]. The yield of polymerization was 98.4% after 40 h (M_n_ = 1750, M_w_/M_n_ = 1.16, initiator efficiency M_calc/_M_n_ = 0.99).

**Scheme 13:**
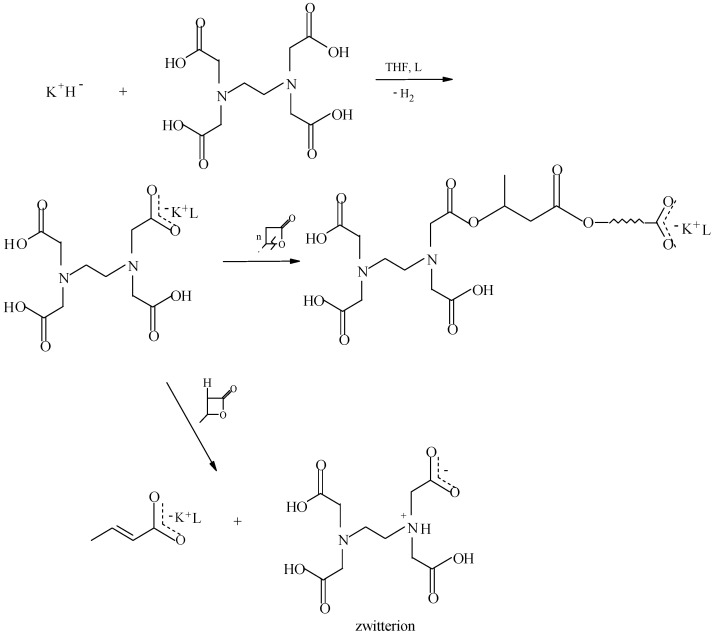
Initiation of BL polymerization with monopotassium salt of ethylenediaminetetraacetic acid, activated by 18C6 (L) [11].

More interestingly, BL polymerization can be initiated with the monopotassium salt of ethylenediaminetetraacetic acid and activated by 18C6 (Scheme 13) [11]. In this case, a star-shaped polyester may form due to a cation exchange reaction. The polymer exhibited a molar mass of M_n_ = 2500, dispersity of M_w_/M_n_ = 1.18, and initiator efficiency of M_calc/_M_n_ = 0.70 (99.2% yield after 40 h).

#### 2.1.4. Two-Electron Transfer Reagents

**Scheme 14:**
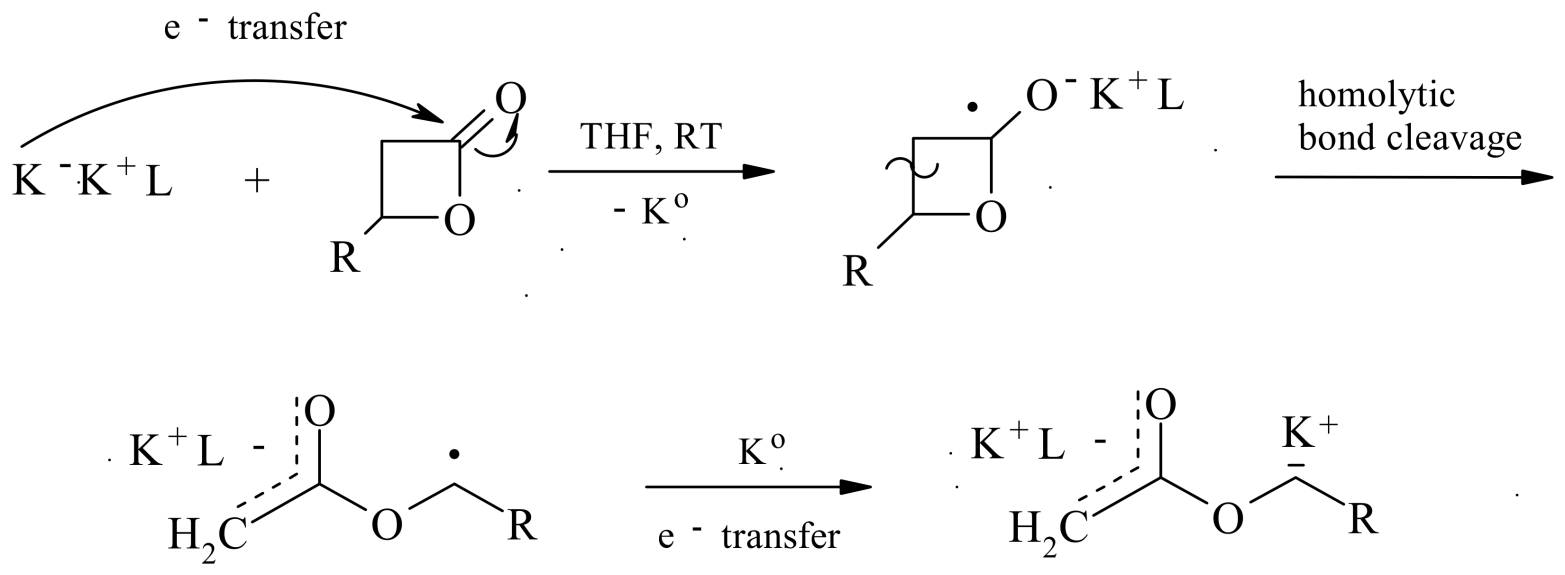
Initiation of PL and BL polymerization with alkalide K^−^K^+^(18C6) via C–C bond cleavage [26]. R is H or Me.

Many studies have been conducted by Jedliński et al. [26,27,28], who treated unusual PL and BL polymerization initiations with alkalide K^−^ K^+^ (18C6). For example, Scheme 14 shows the authors’ proposed mechanism of BL polymerization with potassium anions reacting as an electron transfer reagent [26].

**Scheme 15:**
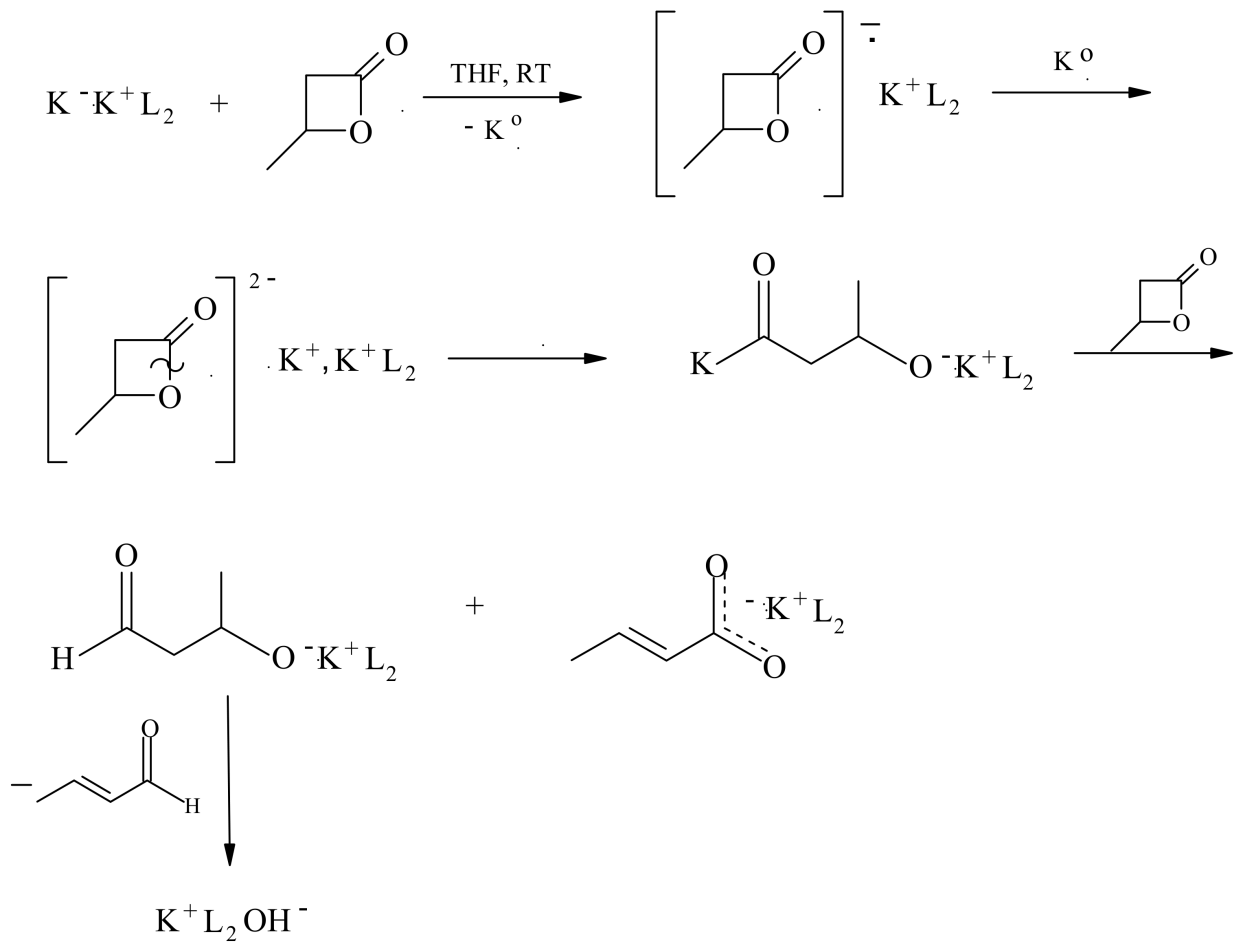
Initiation of β-BL polymerization with alkalide K^−^K^+^(15C5)_2_ via O-acyl bond cleavage [30].

The homolytic C-C bond cleavage was proposed for this system, occurring after the formation of the lactone radical anion. It was proposed that the subsequent reaction with K^o^ derived from K^−^ yielded an enolate carbanion stabilized via resonance, which possesses two types of anionic centers: enolic and carbanionic. It was assumed that the former are inactive, whereas the latter open the monomer ring and begin the polymerization. However, such a reaction course seems improbable. The postulated organopotassium enolate should preferentially deprotonate the lactone molecule, similarly to potassium naphthalenide (Scheme 3), rather than open the lactone ring. Szwarc [29], upon analyzing the mechanism of K^−^ reactions with β-lactones proposed by Jedliński, stated the following: “Interaction of K^−^ solvated by crown ether with β-propiolactone leads to the rupture of the C–C bond instead of the expected opening of the O-alkyl or O-acyl bonds, yielding ethyl acetate eventually. This result calls for verification”. This means that the reaction involved preferentially favors heterolytic rather than homolytic bond scission. Thus, we decided to reinvestigate the unusual ring-opening mechanism of β-lactones. A new corrected mechanism for the initiation step of BL polymerization with potassium anions is proposed in Scheme 15 [30].

Alkalide K^−^ K^+^ (15C5)_2_ was applied instead of K^−^ K^+^ (18C6) because the former has been found to be much more stable than the latter at room temperature [31,32]. The potassium anion is known to react as a two-electron transfer reagent in two steps [33,34,35]. It becomes K^o^ after the first e^−^ transfer from K^−^ to an acceptor molecule, and then potassium forms in the second step of the reaction. We assumed that the studied process initiates with the transfer of a single e^−^ from K^−^ to the LUMO, which is localized mainly between the carbon and oxygen atoms of the lactone carbonyl group, yielding K^o^ and lactone radical anions. Then, K^o^ transfers the second e^−^ to the radical anion. This results in a lactone dianion, which decomposes through heterolytic cleavage of the O-acyl bond. Further reactions mediated by KL_2_OH result in two real initiators of BL polymerization, i.e., potassium 3-hydroxybutyrate and potassium *trans*-crotonate. Note that the same potassium salts form and initiate BL polymerization mediated by KOH or MeOK. These results differ strongly from those presented earlier for the reaction of BL with K^−^ K^+^ (18C6). Therefore, we repeated the experiments using K^−^ K^+^ (18C6) and identified that the same reaction products were identified as when using K^−^ K^+^ (15C5)_2_. We did not observe the formation of the expected derivatives of enolate carbanion derivatives following protonation, methylation, or benzylation, which were postulated for the C–C bond cleavage pathway, along with its reaction product with the monomer [30].

### 2.2. Initiation by Metal-Free Systems (Organocatalysts)

#### 2.2.1. Ionic Salts

**Scheme 16:**
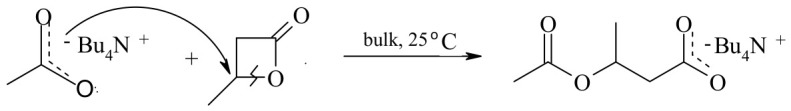
Initiation of BL polymerization with tetrabutylammonium acetate [36].

Kurcok et al. [36] were the first to report that ionic salts of carboxylic acids, such as tetrabutylammonium acetate (TBAA) and tetrabutylammonium hydroxide (TBAH), are more active than the alkali metal salts of carboxylic acid, such as sodium acetate, for BL polymerization. After 500 h, the process, performed in bulk at 25 °C, yielded a polyester with a very high M_n_ = 172,000 and a dispersity of 1.2. The initiation occurred via O-alkyl bond cleavage (Scheme 16) [36]. Chain transfer to the monomer was also observed through the presence of *trans*-crotonate starting groups.

A similar result was obtained during the polymerization of other β-substituted-β-lactones, i.e., β-(methoxymethyl)-β-propiolactone (MOMPL) and β-(ethoxymethyl)-β-propiolactone (EOMPL), initiated with TBAA in THF at 25 °C. However, in the presence of TBA, O-acyl bond cleavage in the BL molecule during the initiation step was established for EOMPL. In all systems, macromolecules with *trans*-crotonate starting groups were also detected as the side product [37]. The yield of the polymerizations was ~90%, and the polymer exhibited a molar mass M_n_(GPC) equal to 1300 and a dispersity of M_w_/M_n_ = 1.3.

**Scheme 17:**
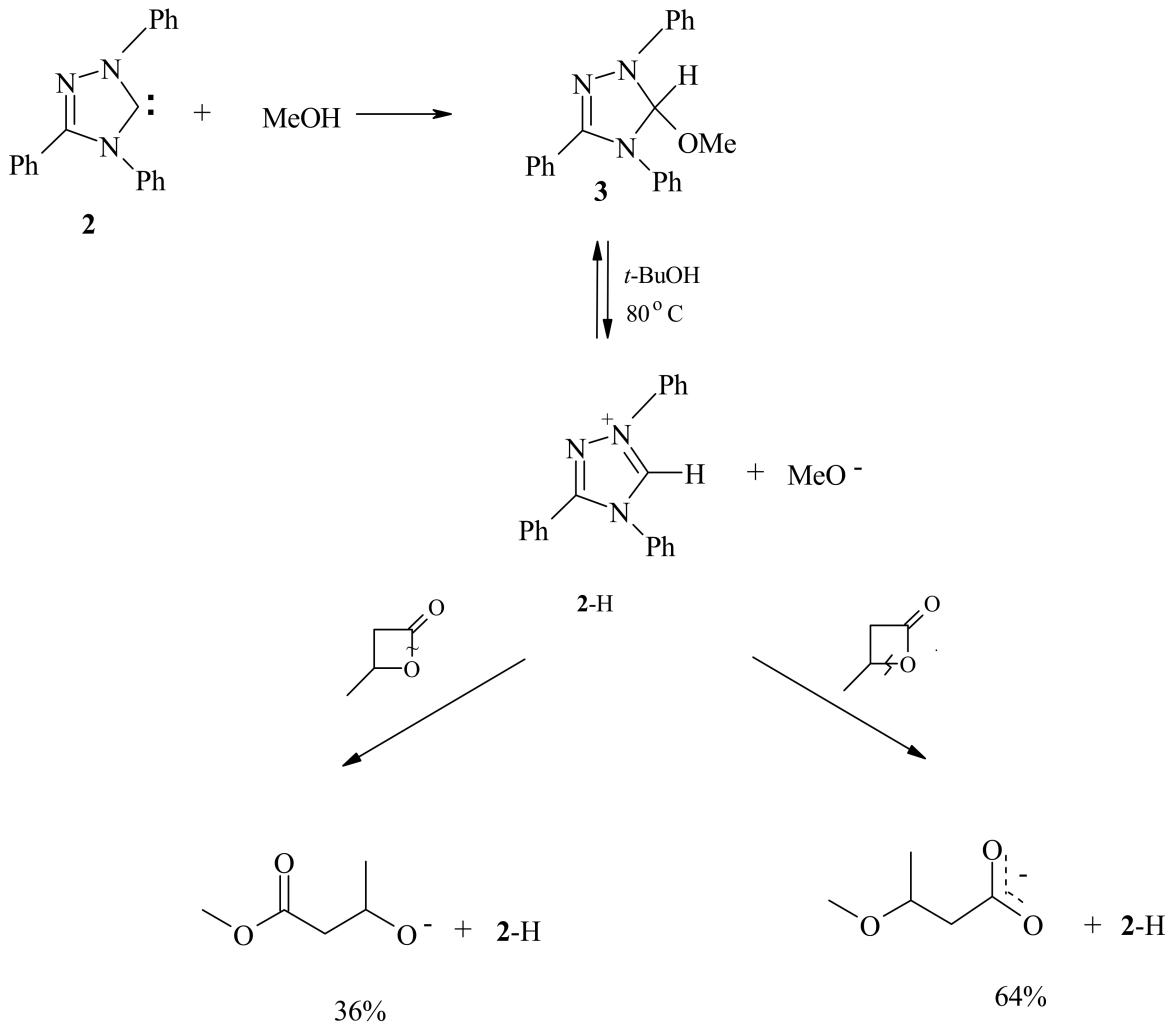
Proposed initiation process of BL polymerization using alkoxytriazolium adduct **3** as latent initiator in *t*-BuOH at 80 °C, implying both O-acyl and O-alkyl cleavage [38].

A high-molar-mass polyester was prepared through the “living” controlled ROP of BL using adduct **2**, which formed from a primary alcohol (MeOH) or carboxylic acid (1-pyrene acetic acid) and a stable carbene, i.e., 1,2,3-triphenyl-4,5-dihydro-1 *H*-1,2,4-triazol-5-ylidene carbene [38]. Structural and kinetic studies indicate that this process is an anionic polymerization occurring with an associated counterion. Furthermore, *t*-BuOH was identified as the ideal polymerization solvent to favor adduct formation and minimize crotonation, which allows for perfect control over molar mass (M_n_ up to 32,000, dispersity (M_w_/M_n_ = 1.1–1.3) and end group fidelity. Interestingly, end-group analysis of the polyester chains revealed the presence of a carboxylic acid end group, confirming that carboxylates were the actual propagating species of the ROP. As active growing centers, both alkoxide and carboxylate groups were found at the early stages of the reaction [38]. However, the relative number of carboxylate end groups increased during the course of polymerization to finally represent the only propagating center for DPs higher than ca. 10. The authors of [38] proposed that anionic polymerization of BL is actually initiated by methoxide anions, where the protonated form of adduct **2** acts as the associated counterion (Scheme 17). A similar phenomenon was previously observed by Penczek et al. [17] for the polymerization of PL initiated with MeOK in DMF solution (Scheme 5).

Application of this new, metal-free anionic initiator allowed for precise control over the nature of the end groups and eliminated any crotonation side reaction.

**Scheme 18:**
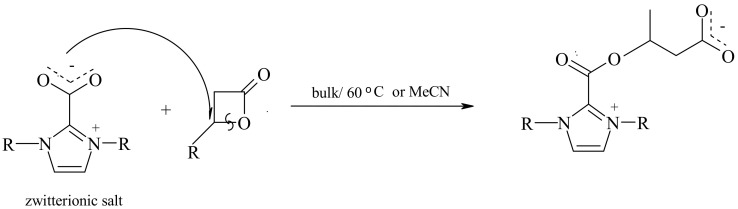
Initiation of BL polymerization with imidazolium carboxylate adduct IMes.CO_2_ [39].

In the specific case of the IMes.CO_2_ adduct, where IMes denotes 1,3-bis (2,4,6-trimethylphenyl)imidazole-2-ylidene), good control was observed in bulk because the experimental M_n_ tracked the calculated values up to 2000. Polymerization occurs faster in polar solvents, such as DMSO and MeCN, than in lower-polarity solvents such as THF or toluene at 60 °C. It was observed that O-alkyl bond cleavage occurred during the initiation step (Scheme 18) [39].

Other ionic initiators (i.e., phosphazene-based carboxylic salts) have also been used for the anionic polymerization of BL [40]. Regarding ammonium-based carboxylate salts, they demonstrated increased activity when the interaction between the carboxylate anion of 1-pyrene acetic acid and its protonated phosphazene counterion (for example, from *t*-butylimino-tris(dimethylamino)phosphorane as phosphazene base) is weakened via ion separation. During polymerization, a transfer reaction to the monomer occurred. However, the perfectly controlled and “living” process using these initiators for the polymerization of α,α-protected BL was reported to eliminate any possible proton elimination reaction [41].

#### 2.2.2. Neutral Bases

**Scheme 19:**
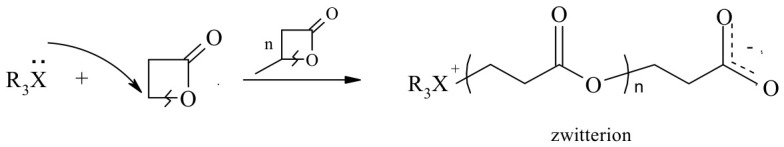
Polymerization of PL initiated with tertiary amines or phosphines [17]. X is N or P.

The only group of initiators for which a unanimous opinion prevails comprises tertiary amines and phosphines, which lead to the zwitterionic polymerization of PL and BL [17,42,43,44]. It is generally accepted that, in the zwitterions formed, the anionic centers are carboxylate anions (Scheme 19) [17].

**Scheme 20:**
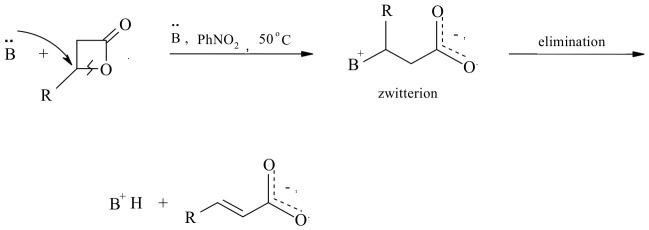
Initiation of β-lactone polymerization with a weak base via ring-opening followed by elimination of the protonated base [9]. R is H or Me, and B denotes pyridine.

Kricheldorf et al. [9] investigated several neutral bases as initiators of BL polymerization. The basicity (b) of the initiators was varied over a wide range, between p*K*_a_ *≈* 5 for pyridine and p*K*_a_ *≈* 11 for triethylamine. Another important factor is their nucleophilicity (n), which is responsible for the efficiency of the nucleophilic attack at the β-carbon of the lactone (Scheme 20) [9].

**Scheme 21:**
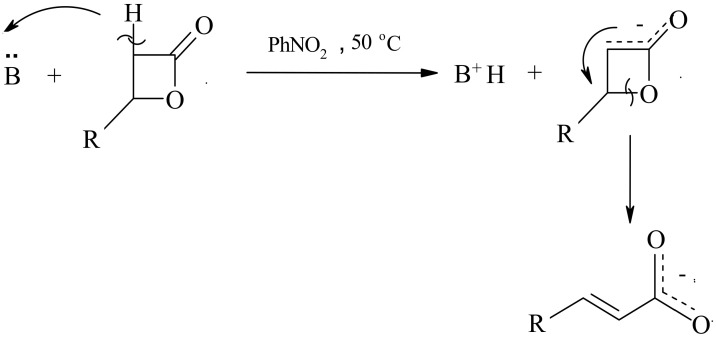
Initiation of β-lactone polymerization with a strong base via direct deprotonation [9]. R is H or Me, and B denotes triethylamine.

The following qualitative order of the n/b ratio is expected: triphenylphosphine > pyridine > *N*-methylmorpholine > triethylamine. The formation of ionic reaction products from neutral bases is favored by a higher dipole moment of the reaction medium, e.g., in nitrobenzene. Interestingly, pyridine yielded the highest concentration of crotonate groups, and the strongest base (i.e., triethylamine) yielded the lowest concentration. Even the initiator with the highest n/b (i.e., triphenylphosphine) produced a sizable concentration of crotonate groups. The authors postulated that the deprotonation mechanism presented in Scheme 20 is favored for a weak base [9]. This results from the nucleophilic attack at the β-carbon followed by the elimination of the protonated base. This deprotonation is base-promoted. A similar deprotonation mechanism has been postulated by Yamashita et al. [45] to explain the high yields of acrylate groups in pyridine-initiated polymerization of PL. Conversely, it is unlikely that a sterically hindered base, such as triethylamine, reacts in the same manner. In this case, direct deprotonation of the monomer does occur through a nucleophilic attack at the β-carbon, followed by the formation of lactone enolate, as shown in Scheme 21 [9].

Hence, it is obvious that two deprotonation mechanisms exist, and the n/b of the initiator indicates which one prevails [9]. Using moderate bases, such as 4-(*N*,*N*-dimethylamino)pyridine, 4-methylpyridine, or pristine pyridine, for the polymerization of PL and BL [9], the complete elimination of pyridinium ions and the formation of acrylate and crotonate starting groups were observed, respectively [9,45]. However, the polymerization of β-pivalolactone proceeds via zwitterionic ring-opening polymerization in the absence of cyclic structures [45].

**Scheme 22:**
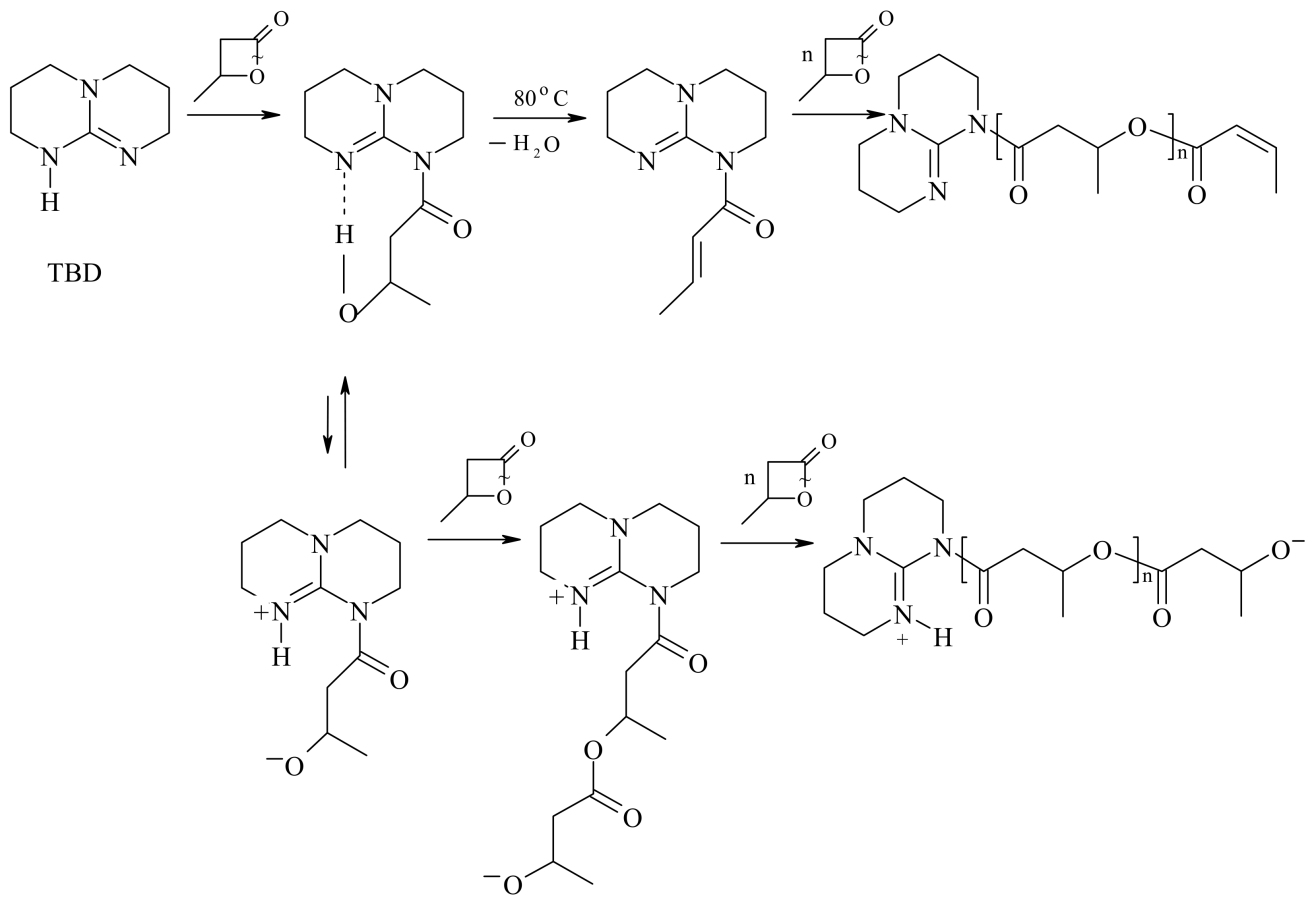
Possible initiation mechanism (i.e., nonionic and zwitterionic) in BL polymerization promoted by guanidine (TBD) [46].

Interestingly, Jaffredo et al. [46] reported that under specific reaction conditions, basic organocatalysts of the guanidine, amidine, and phosphazene type effectively polymerize BL in bulk at 60 °C. The prepared polymers exhibit controlled molecular features—namely, predictable molar masses, narrow dispersities, and well-defined functional end groups. The prevailing mechanism of the process involves several steps—namely, the formation of a guanidine–BL adduct, dehydration of this adduct to the corresponding *N*-acyl-α,β-unsaturated species, and subsequent propagation through a “living” process (Scheme 22).

An alternative mechanism can be envisioned to account for the end-capping of the polymer chains by the organic initiator, which applies to guanidine and the two other non-functionalized bases, i.e., amidine and phosphazene [46]. It would involve the partial dissociation of an adduct into a zwitterionic form, which would ring-open a BL moiety. Following this second insertion, the resulting zwitterion would persist and then propagate. The observed crotonate end groups would form through the dehydration of the propagating species, thus acting as a termination pathway. However, further work is needed to draw a definitive conclusion about the polymerization course [46].

**Scheme 23:**
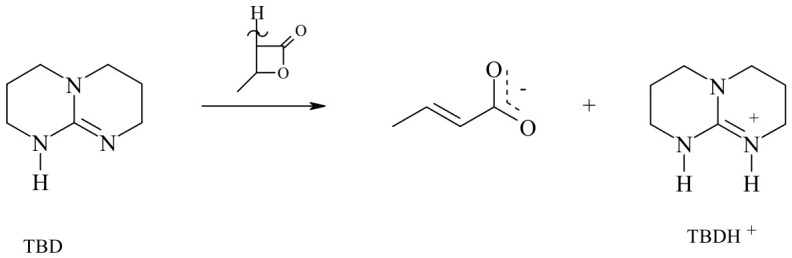
Corrected mechanism of initiation of BL polymerization with guanidine (TBD) [47].

Recently, a reinvestigation of the mechanism [47] indicated that unprotected β-lactones, such as BL, are obtained mainly via anionic polymerization involving the in situ formation of crotonate species (Scheme 23) and minorly by the nucleophilic route proposed previously.

A similar effect is observed in the polymerization of β-substituted-β-PLs (β-PL^OR^, where R can be All, Bn, n-Bu, or SiMe_2_*t*-Bu) initiated with phosphazene (BEMP) [48]. On the contrary, the highly nucleophilic guanidine (TBD) forms an N-acyl-α, β-unsaturated adduct, which subsequently propagates through the O-acyl bond cleavage of the β-PL^OR^. Finally, the observed dual basic and nucleophilic activity of amidine (DBU) favors the scission of both O-alkyl and O-acyl bonds of the β-PL^OR^.

Anionic polymerization of β-lactones is frequently treated as “living” due to the lack of chain transfer reactions to the polymer (transesterification) and thus, practically, to the monomer. A characteristic feature of the anionic polymerization of β-lactones is the deprotonation of the monomer, which is mediated mainly by the initiator. This reaction results in crotonization, which is undesired as it leads to Michael additions, resulting in a loss of control in the polymerization, decreased molecular masses, and sometimes even catalyst poisoning.

**Scheme 24:**
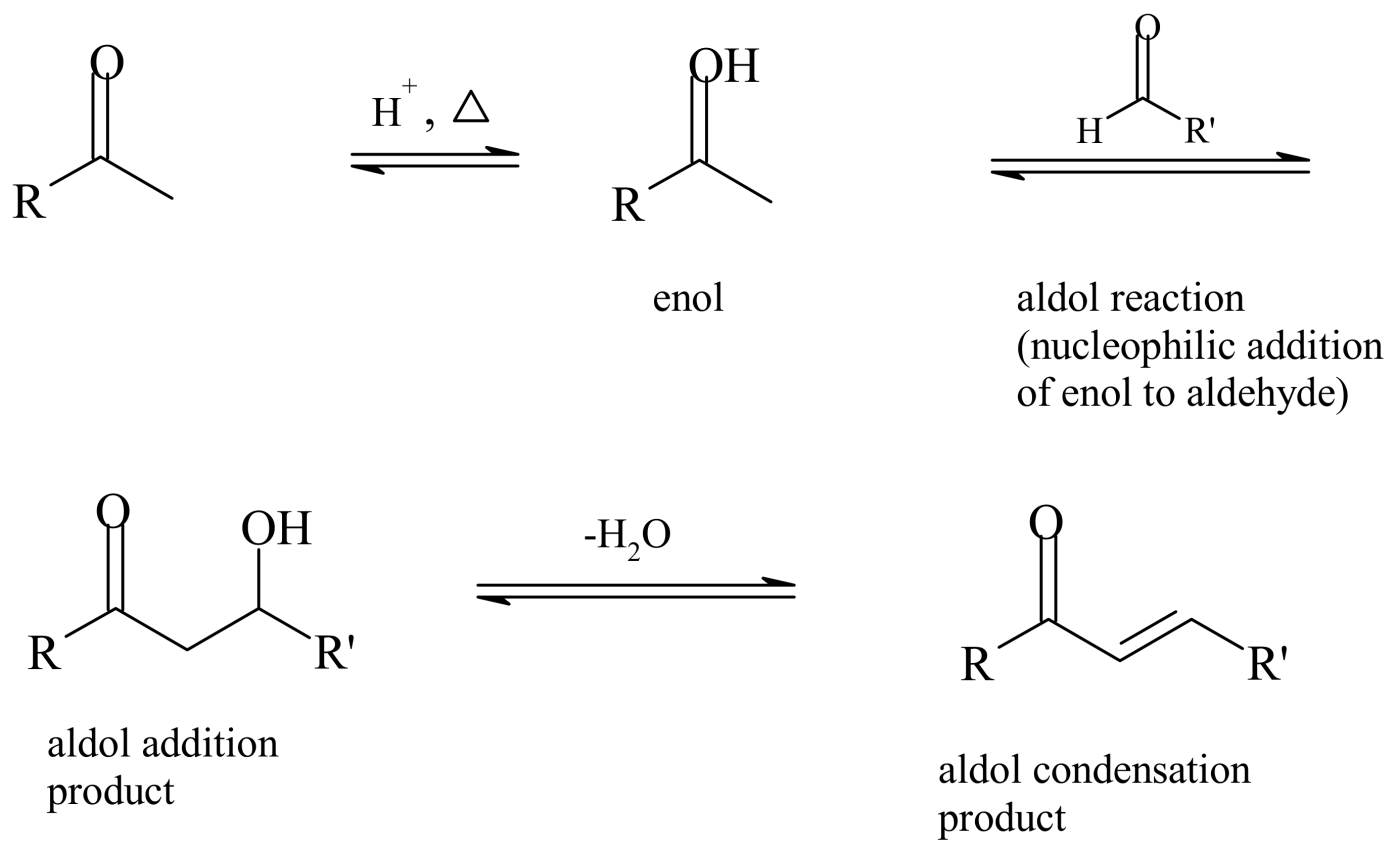
Exemplary aldol condensation involving the reaction of a ketone with an aldehyde, catalyzed by an acid (ENOL mode) [48].

Note that, in organic chemistry, crotonization refers to the dehydration stage within the aldol condensation of aldehydes or ketones. One of them must possess at least one H-atom at the C-atom joined with the –CHO or >C=O group. Reactions are catalyzed by acids or bases. The resulting Β-hydroxyl aldehyde or β-hydroxy ketone loses a molecule of water at elevated temperatures to form an α,β-unsaturated carbonyl compound. This step is driven by the formation of a stable conjugated system (C=C-C=O), e.g., crotonaldehyde (Scheme 24).

Aldol condensation is a fundamental part of building complex carbon skeletons in synthetic chemistry, particularly for generating stable conjugated systems.

## 3. Cationic ROP

### 3.1. Salts of Complex or Noncomplex Anions (Counterions)

#### 3.1.1. Electrophilic Reagents

**Scheme 25:**
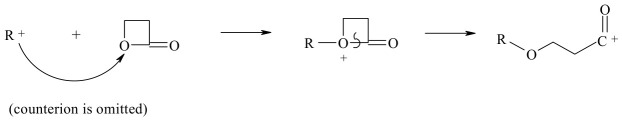
Mechanism of cationic PL polymerization via O-acyl bond cleavage [49].

Many authors, such as Ito et al. [49], have proposed mechanisms for the cationic polymerization of PL initiated with alkyl or acyl cations (R^+^). A general mechanism of initiation involves an attack by the initiating cation on the endocyclic oxygen atom of the monomer, followed by exclusive cleavage of the O-acyl bond. Polymerization proceeds with acylium cations as active centers (Scheme 25) [49].

Furthermore, Ito et al. [50] showed that the lower dissociation energy of the O-alkyl bond in esters, compared to the O-acyl bond, should result in preferred O-alkyl bond cleavage.

Later, ionic dihalonium salts (Me_2_Br^+^SbF_6_^−^ or MeI^+^SbF_6_^−^) and acyllium salts (MeCO^+^SbF_6_^−^ or EtCO^+^SbF_6_^−^) were used by Penczek et al. [51] as alkylating or acylating reagents. These salts possess a complex hexafluoroantimonate anion, which is not able to form covalent bonds since its coordination sphere cannot be enlarged further.

**Scheme 26:**
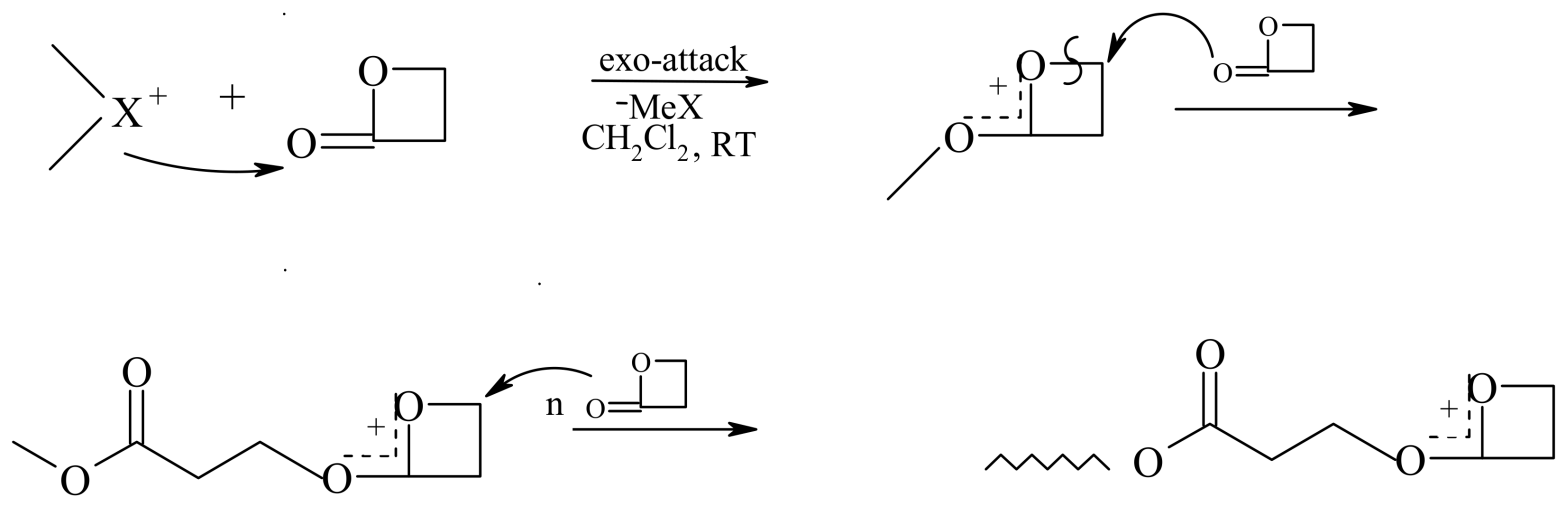
Mechanism of cationic PL polymerization via O-alkyl bond cleavage [50]. X is Br or I (counterion is omitted).

The results of the studies on polymerization mediated by dihalonium salts indicated that both initiation and propagation via electrophilic attack by the carbenium cation occur exclusively at the exocyclic oxygen atom of the monomer molecule and that monomer addition to the active tertiary oxonium center proceeds with O-alkyl bond cleavage (Scheme 26) [50].

**Scheme 27:**
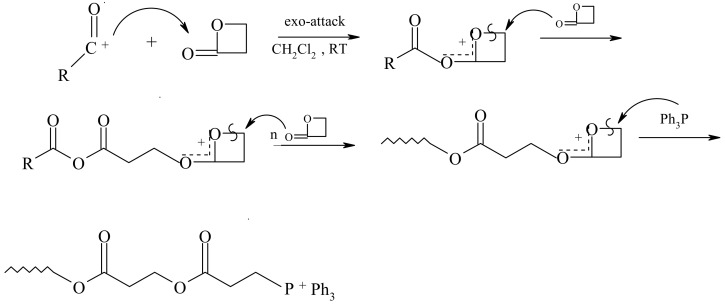
Attack by the acylium cation on the exocyclic oxygen atom, resulting in O-alkyl bond cleavage [50]. R is Me or Et.

Moreover, the initiation of PL polymerization with acylium cations of hexafluoroantimonates—namely, MeCO^+^SbF_6_^−^ and EtCO^+^ SbF_6_^−^—is more complex. Based on the analysis results of terminal groups in polymers, Penczek et al. [50] proposed that during initiation (and, presumably, also the first propagation steps) acylium cations participate in polymerization by attacking both the exo- and endocyclic oxygen atoms in almost equal proportions. The attack on the exocyclic oxygen atom leads to the formation of active oxonium centers regenerated in the subsequent propagation steps (Scheme 27) [50].

**Scheme 28:**
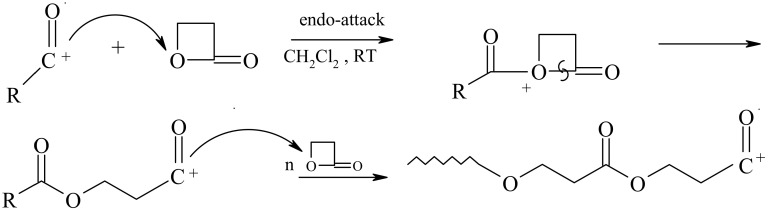
Attack by the acylium cation on the endocyclic oxygen atom, resulting in O-acyl bond cleavage [50].

Furthermore, the attack by the endocyclic oxygen atom leads to the regeneration of active acylium centers (Scheme 28) [50].

**Scheme 29:**
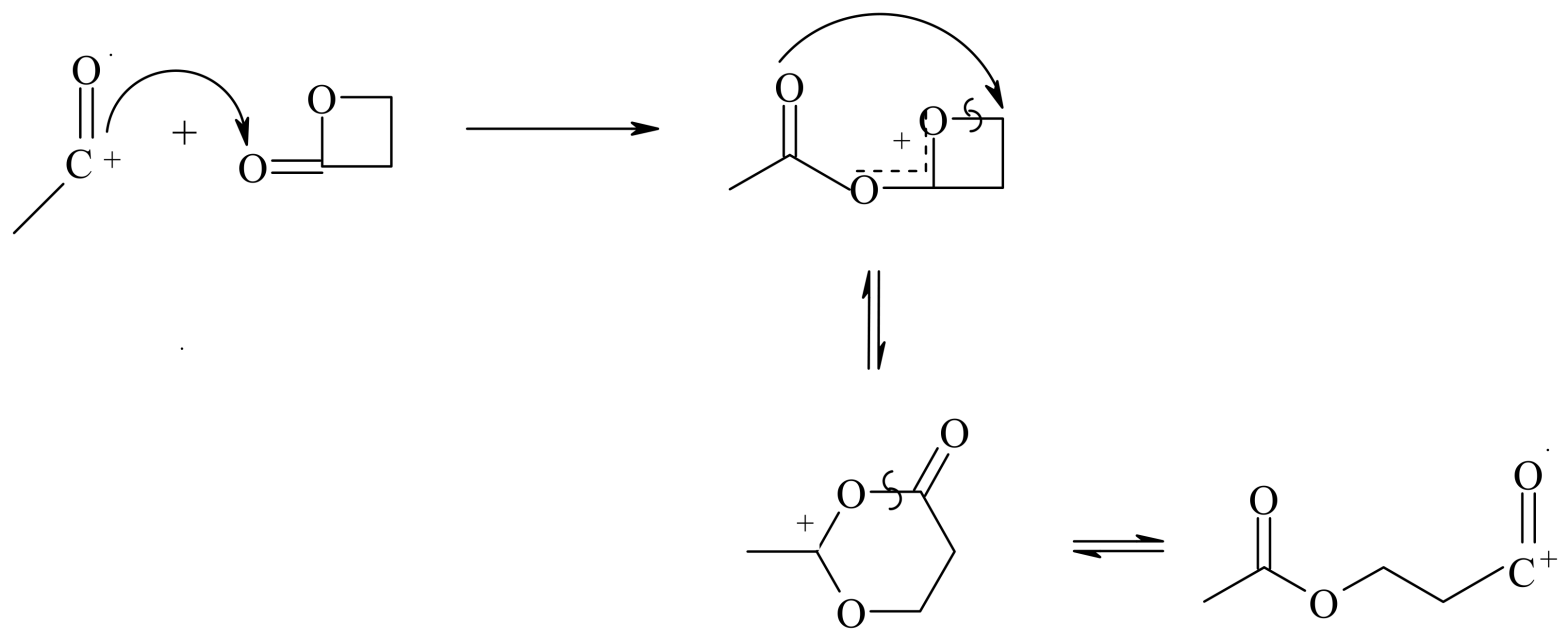
Formation of acetate initial groups through intramolecular alkylation of anhydride groups [52].

**Scheme 30:**
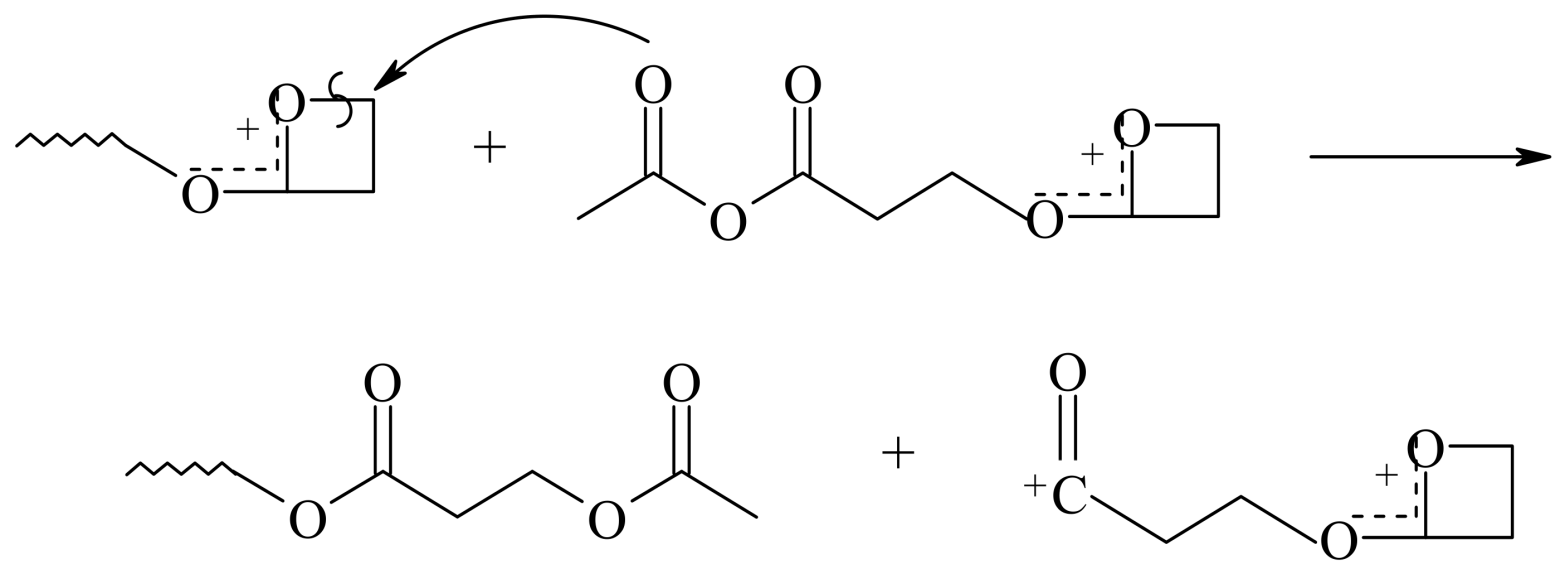
Formation of acetate end groups through intermolecular alkylation of anhydride groups [52].

However, at each propagation step, approximately half of the acylium cations are converted into active oxonium centers. Thus, after ≈10 monomer additions to the set of macromolecules initiated by acylium cations, oxonium cations constitute more than 99.9% of the active species [50]. The triphenylphosphine test exclusively indicated the presence of alkylating chain ends. By analyzing this interesting mechanistic scheme, Kricheldorf et al. [52] concluded that this mechanism relies on unproven speculations. The first of these is the lack of selectivity of the acylium ion; the second is the assumption that the product of endocyclic attack undergoes propagation faster than transacylation. Intra- or intermolecular migration of the acetylium ion to a more nucleophilic and more basic exocyclic oxygen is much more likely. The question remains as to how the formation of acetate groups can be explained if the acetylium ion exclusively attacks the exocyclic oxygen or migrates to an exocyclic oxygen before subsequent reaction steps occur. It was proposed that anhydride starting groups are alkylated either in an intramolecular (Scheme 29) or intermolecular manner (Scheme 30) [52].

Interestingly, Kricheldorf et al. [52] studied the polymerization of PL initiated by means of methyl trifluorosulfonate (MeOSO_2_CF_3_) and acetylium perchlorate (MeCOClO_4_) in PhNO_2_ at 50 °C. The polymers obtained possessed alkyl ester and acetate starting groups, respectively, indicating chain growth via O-alkyl cleavage of the lactone. In order to explain the mechanism of this unusual polymerization, the authors proposed that initiators dissociate in the reaction mixture, yielding anions and cations capable of initiation. However, ^13^C NMR analysis did not confirm such a phenomenon. Thus, the equilibrium of reactions (1) and (2) must be shifted far to the left.

MeSO_3_CF_3_ ⇄ Me^+^ + CF_3_SO_3_^−^ (1)

MeCOClO_4_ ⇄ MeCO^+^ + ClO_4_^−^ (2)

Moreover, Penczek et al. [7] stated that during cationic ROP, noncomplex anions such as MeSO_3_^−^, CF_3_SO_3_^−^, FSO_3_^−^, and ClO_4_^−^ form covalent bonds by sharing two electrons from an oxygen atom to form a sigma bond. Therefore, whenever polymerization proceeds with these noncomplex anions, reversible polyester formation may occur.

The results of the works of Penczek et al. [50] and Kricheldorf et al. [52] led to the conclusion that cationic polymerization of PL initiated by carbenium or acylium cations proceeds almost exclusively via O-alkyl bond cleavage of the lactone, which involves an electrophilic attack on the exocyclic oxygen of the monomer during the initiating step. This is because the exocyclic oxygen is much more nucleophilic than the endocyclic one, and the delocalization of the positive charge strongly stabilizes the cation formed [51]. Yamashita et al. [45] also reported that β,β-dimethyl-PL is much more reactive in cationic polymerization than unsubstituted PL. The monomer also polymerizes via O-alkyl bond fission, featuring an oxonium ion as the growing chain end.

**Scheme 31:**
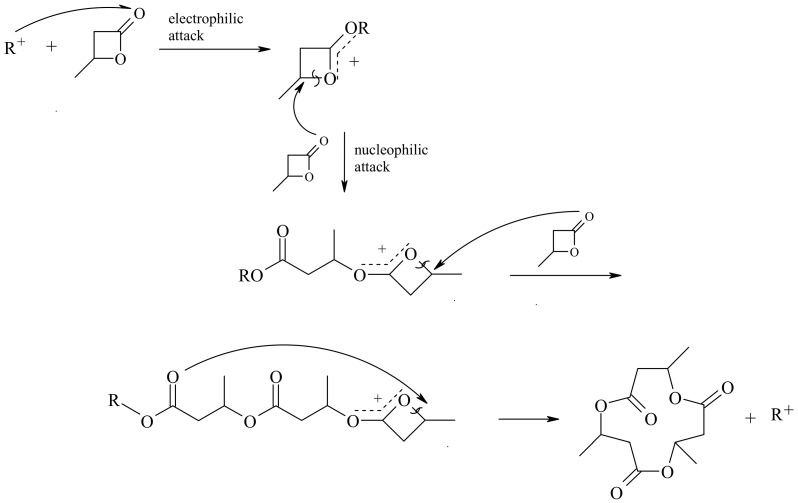
ACE mechanism in cationic β-lactone polymerization initiated with an electrophilic ionic reagent involving the formation of a cyclic structure. R is H, alkyl, or acyl (counterion is omitted).

In general, the principal propagation of the cationic ROP of β-lactones initiated with electrophilic ionic reagents involving protonic acids [52] is called the active chain-end (ACE) mechanism [53]. Propagation proceeds via nucleophilic attack by the exocyclic oxygen atom of the monomer molecule on the β-carbon of the resonance-stabilized tertiary oxonium ion located at the growing chain end. The polymer chain is formed via O-alkyl bond cleavage and the process eventually leads to the formation of cyclic PBL by end-to-end closure (Scheme 31), which indicates that this polymerization is not “living”.

#### 3.1.2. Hydride Anion Acceptors

**Scheme 32:**
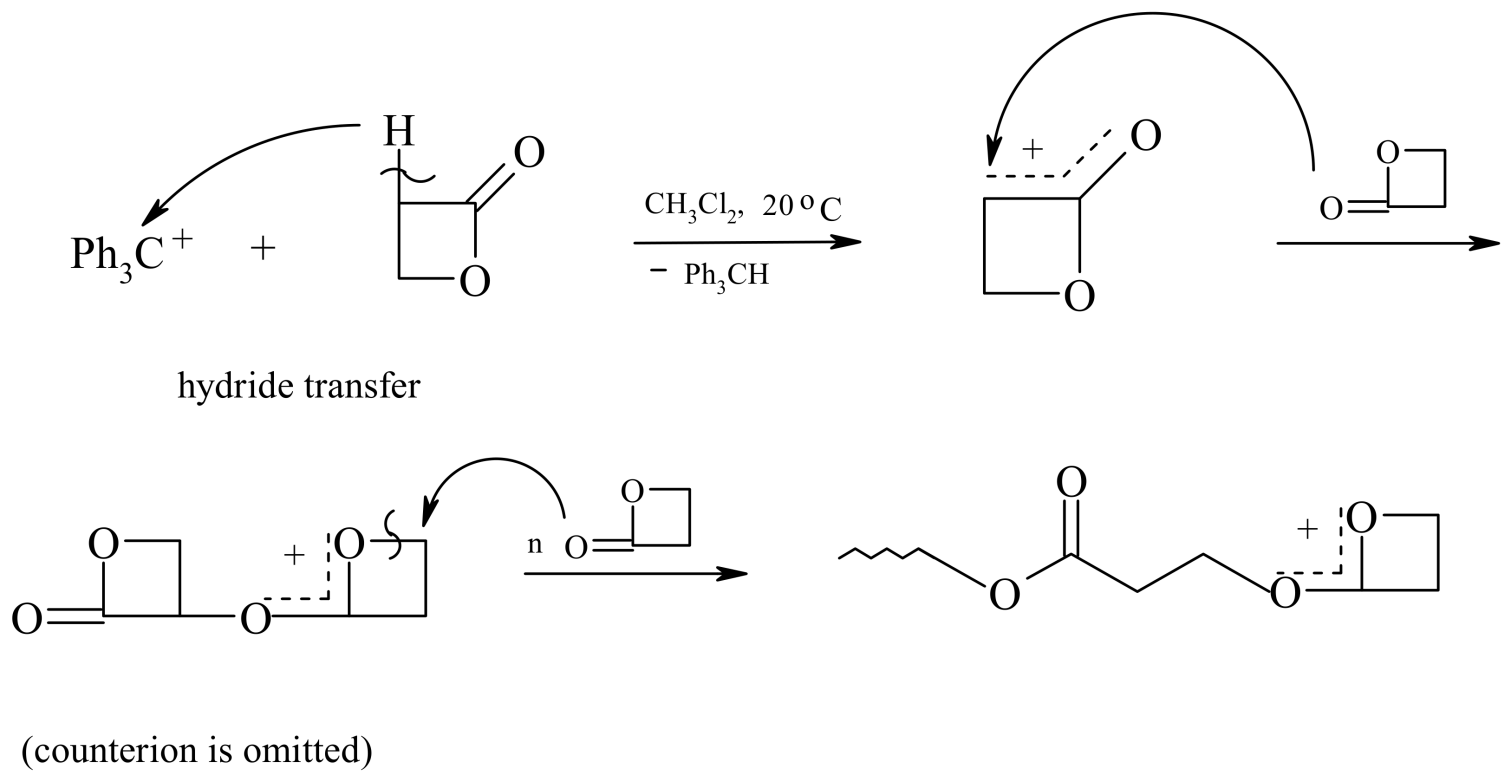
Polymerization of PL initiated with tritylium salts via hydride anion transfer from monomer (donor) to initiator (acceptor).

Khomyakov et al. [54] reported that, in the presence of tritylium salts, slow initiation of PL polymerization can occur. Based on ^1^H NMR spectra of the polymers obtained, the authors excluded the possibility of an attack by the tritylium cation on either the exocyclic or endocyclic oxygen atom of the monomer molecule. Examination of the spectrum after polymerization in bulk over Ph_3_C^+^SbCl_6_^−^ indicated that, in this specific case, initiation proceeds through a nucleophilic hydride anion transfer from the monomer to the initiator [54]. Almost all of the tritylium salt is converted into triphenylmethane in this reaction. Then, the formed carbocation reacts with the next monomer molecule, yielding a product with an acylium cation end group, which attacks the exocyclic oxygen atom. The starting groups in the polymer were not determined. However, we proposed a much more probable course of PL polymerization without the formation of an intermediate acylium cation (Scheme 32). Propagation occurs due to the ACE mechanism in this system.

It has also been observed [54] that the type of counterion does not significantly affect the rate of the trityl salt reaction with PL. A study on the solution polymerization of PL in CH_2_Cl_2_ or PhNO_2_ at relatively low monomer concentrations showed that the hydride transfer was complete. Depending on the experimental conditions, some residual Ph_3_C^+^ content was detected in both solvents after the polymerization. This effect is probably due to the participation of triphenylmethane in the reaction with the reactive centers. It could be that the tritylium ions are formed in the system.

### 3.2. Electrophilic Reagent/Hydroxylic Compound Systems

**Scheme 33:**
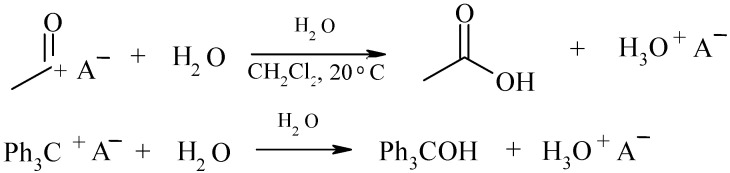
Formation of oxonium salts with different counterions in reactions of cationic initiators with excess water [55]. A^−^ is SbCl_6_^−^, SbF_6_^−^, AsF_6_^−^, PF_6_^−^, or BF_4_^−^ (complex anions).

An interesting case concerning the cationic polymerization of PL with various initiators in the presence of hydroxylic compounds, such as water or alcohols, was reported by Khomyakov et al. [55]. The initial reactions in these systems mediated with water are shown in Scheme 33 [55]. 

**Scheme 34:**
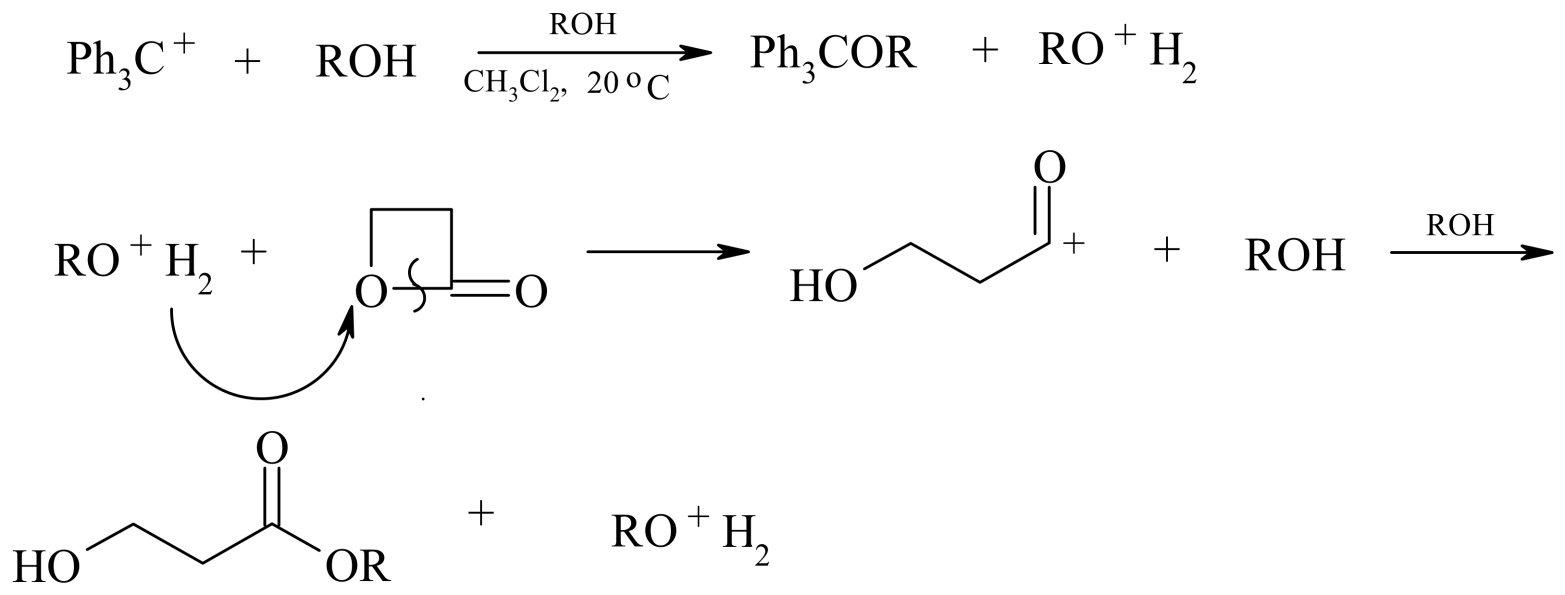
Initiation of PL polymerization with tritylium salts in the presence of alcohol via O-acyl bond cleavage [55].

**Scheme 35:**
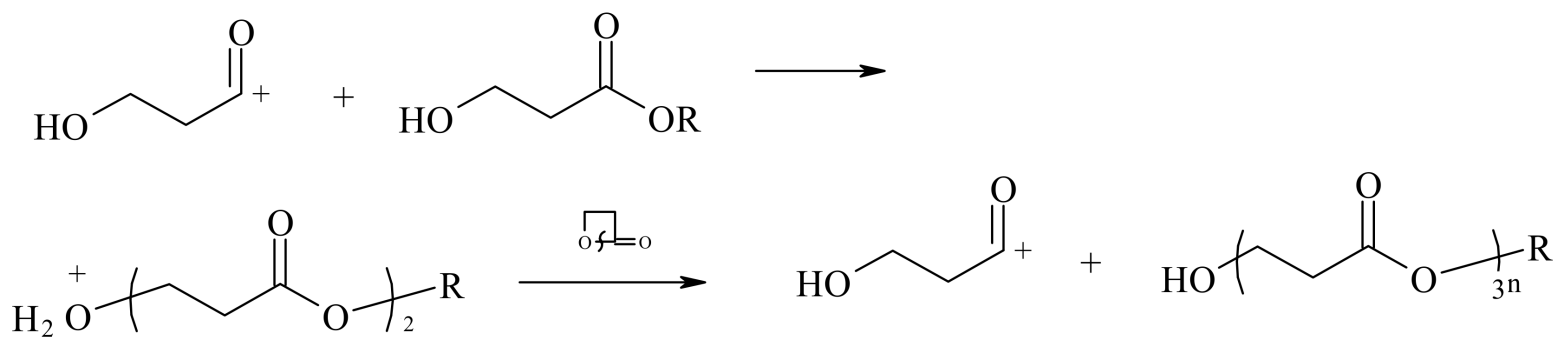
Growth of polymer chain through transfer to monomer via O-acyl bond cleavage [55].

Note that water and alcohol behave almost identically in this process. The mechanism of PL polymerization was called the “hydroxo–mechanism” and essentially consists of polymerization initiation by oxonium salt and growth of the polymer chain through a transfer to the monomer (Scheme 34 and Scheme 35) [49]. Considering this mechanism, Penczek et al. [50] assumed that initiation involving protonic acids can result in unique characteristics [56,57].

The effect of the counterion’s nature was examined using tritylium salts with various counterions in the presence of water. The results show that the counterion has a strong effect on the polymerization rate. It may be assumed that the SbCl_6_^−^, SbF_6_^−^, and AsF_6_^−^ counterions promote complete dissociation of the propagation center into free-O^+^H_2_ ions, which carry on the process [49].

### 3.3. Acid/Alcohol Systems

**Scheme 36:**
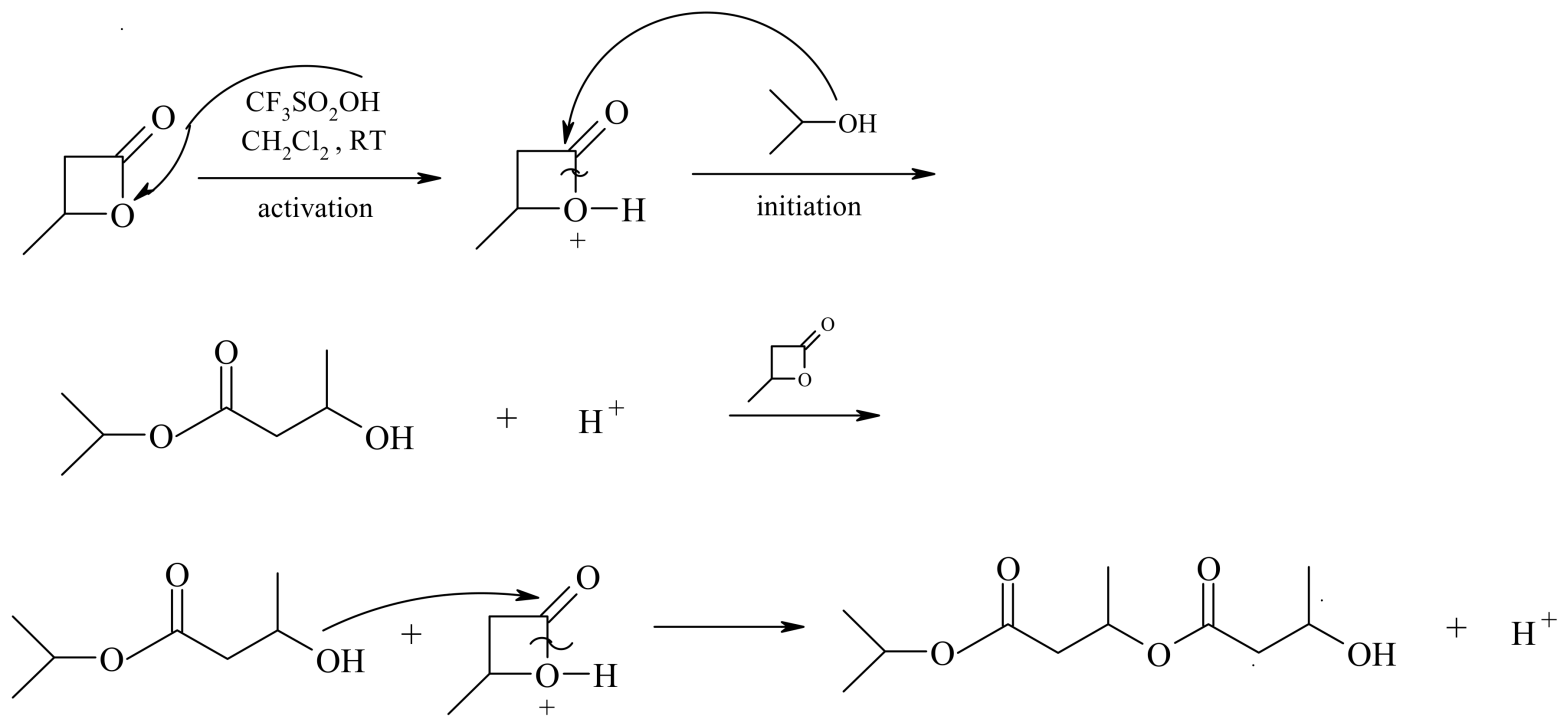
Cationic polymerization of BL occurs through the AM mechanism (in the first step, H^+^ attacks the endocyclic oxygen atom in the monomer) [53].

In 2013, Basko et al. [53] presented an excellent explanation of cationic polymerization of BL performed in CH_2_Cl_2_ at room temperature, with CF_3_SO_2_OH as the catalyst and isopropanol as the initiator. In this case, the cationic mechanism is complete; it proceeds via an activated monomer pathway, in which propagation occurs through an attack by the oxygen atom of the terminal hydroxy group on the α-carbon in the protonated monomer molecule. Such a mechanism is known as the activated monomer (AM) mechanism. The reaction pathway proposed by the authors is shown in Scheme 36 [53].

**Scheme 37:**
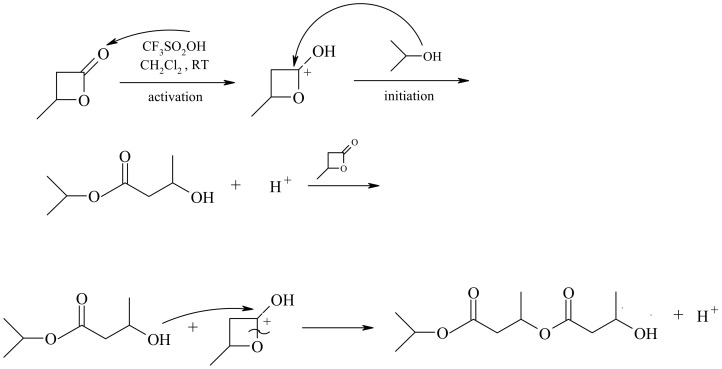
Alternative AM mechanism of BL cationic polymerization (in the first step, H^+^ attacks the exocyclic oxygen atom in the monomer).

However, we proposed a more probable mechanism of activation involving a proton attack on the more nucleophilic and basic oxygen atom of the carbonyl group (Scheme 37).

Note that, in both cases, propagation occurs due to a nucleophilic attack on an oxygen atom of the terminal hydroxy group on the same carbon atom in the protonated monomer molecule, resulting in the formation of the same polymer chains via O-acyl bond cleavage. If the growth of the polymer occurred according to the AM mechanism, each growing macromolecule should contain a starting group from the used alcohol. For [BL]_o_/[HO-]_o_ ratio < 30, all macromolecules are linear. However, for higher [BL]/[HO-] ratios, such as when this ratio is equal to 125, a low molar weight was observed in the SEC chromatogram of the polymer (~4%) despite slow monomer addition. Analysis of the MALDI–TOF spectra indicated that this fraction consisted of cyclic macromolecules. Thus, it was suggested that the reaction of the protonated monomer with another monomer molecule instead of an alcohol occurs with subsequent propagation involving tertiary oxonium ions, with the ACE mechanism eventually leading to the formation of cyclic oligomers by end-to-end closure [58]. These polymers exhibited M_n_ = 2300–5900, M_w_/M_n_ = 1.3–1.4, and a yield of ~98%. Similar results were obtained by Bourissou et al. [59], who used MeSO_2_OH or CF_3_SO_2_OH acids as catalysts and *n*-pentanol as the initiator.

With the CF_3_SO_2_OH/MeOH system, it was possible to obtain PBL with M_n_ = 4600 and M_w_/M_n_ = 1.12 [57]. O-acyl bond cleavage was postulated during the propagation. The polymerization of chiral BL proceeds with the retention of the configuration from (*R*)-β-butyrolactone.

It is worth noting that the cationic polymerization of BL mediated by metal-free acid/alcohol systems represents a valuable alternative to polymerization using metal-based initiators, as it is more environmentally friendly.

## 4. Coordinative (Pseudoanionic) ROP

### 4.1. Initiation with Simple Homoleptic Metal Complexes

**Scheme 38:**
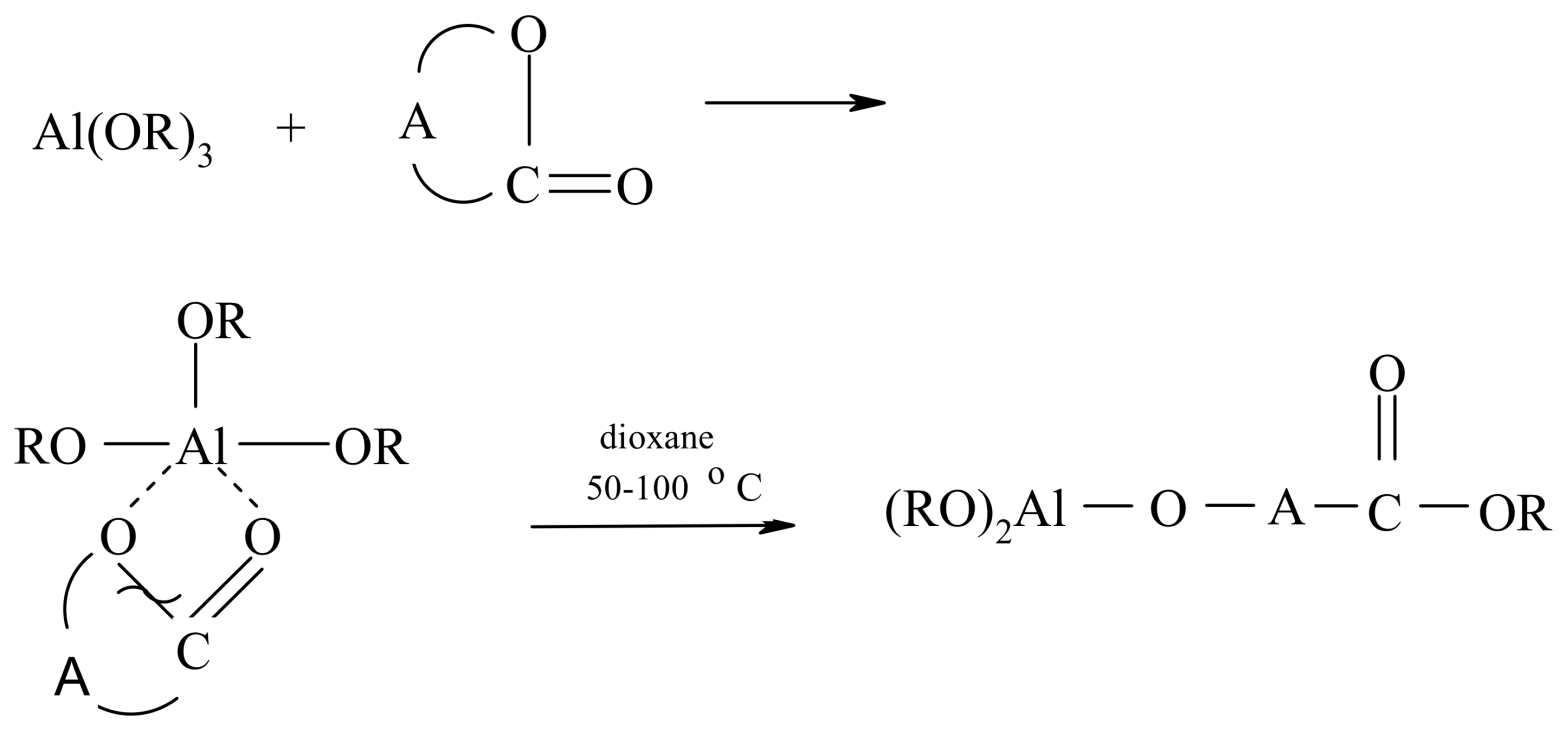
Initiation of PL polymerization with aluminum alkoxide [60].

In 1975, Teyssié et al. [60] demonstrated that aluminum isopropoxide initiates the polymerization of PL in such a way that the O-acyl bond of β-lactone is cleaved (Scheme 38) and not the O-alkyl bond. It is characteristic of this mechanism that the alkoxide group of the initiator forms the dead end of the growing chain end. Only one alkoxide group per aluminum is active as an initiating species.

Subsequently, Kricheldorf et al. [61] polymerized PL and BL in the presence of various covalent metal alkoxides, i.e., (*n*-Bu)_3_SnO, MeAl(O-*i*-Pr)_3_, Ti(O-*n*-Bu)_4_, Zn(O-*n*-Bu)_4_, and Zr(O-*i*-Pr)_4_. The polymerizations were not “living” processes due to slow initiation and transesterification reactions, especially at higher temperatures. NMR end-group analysis clearly indicated that all covalent metal alkoxides yielded alkyl ester end groups and never alkyl ether groups. The results suggest that both the initiation and propagation steps of the polymerization have a uniform mechanism, which is characterized by the cleavage of the O-acyl bond.

The proposed nonionic insertion–coordination mechanism is characteristic for all metal alkoxides or phenoxides with free *p* or *d* orbitals and results in the absence of *trans*-crotonate groups in the polymers obtained. The differences between ionic and covalent initiators, even those sharing an alkoxide structure, arise because the latter are much weaker bases than alkoxide anions, whether paired or free [14]. Studies on initiation with covalent alkoxides have consistently revealed no products other than the addition products of the initiator and monomer. The first step of the reaction involves the complexation of the β-lactone at the carbonyl oxygen, which is the most basic and nucleophilic site of a lactone. IR spectroscopic evidence for such complexation has been presented by Kohn et al. [62], who used various tin and aluminum compounds as complexing reagents. The next step involves the cleavage of the acyl–oxygen bond of the lactone through the insertion of the lactone into the weakly polarized metal–oxygen bond [61]. Other alkyltin derivatives, such as (*n*-Bu)_2_Sn(OMe)_2_, *n*-BuSn(OMe)_3_, [(*n*-Bu)_3_Sn]_2_O, and (Ph_3_Sn)_2_O, have been investigated further, demonstrating overall performance similar to that described for (*n*-Bu)_3_SnOMe [63,64,65,66,67,68]. However, the nature of the end groups and the actual mechanism operative in these tin-promoted polymerizations have not yet been fully clarified. While NMR analysis has revealed only OH and CO_2_Me end groups in polyesters produced with (*n*-Bu)_2_ Sn(OMe)_2_, both COOH and CO_2_Me end groups have been detected for polymers obtained through the ROP of *rac*-BL with (*n*-Bu)_3_SnOMe. This suggests that polymerization in the latter case is not solely initiated by an insertion mechanism into the Sn–OMe bond [63]. These compounds are the first class of initiators yielding more or less syndiotactic polyesters. Kricheldorf et al. [64] showed that the stereoselectivity of the *rac*-BL polymerizations slightly increases with the steric hindrance of the catalysts. Furthermore, two distannoxane initiators, which allow for better control of the polymerization, have been shown to produce a predominantly syndiotactic PBL [67]. A similar initiation course was proposed by Save et al. [69] for PL polymerization initiated by La(O-*i*-Pr)_3_. However, in this case, small NMR signals corresponding to acrylate groups (ca. 18%) were observable, indicating that side elimination occurred simultaneously with the predominant normal propagation process. The La–O bond should instead be considered a highly polarized covalent bond. The reactivity of the La–O bond is much higher than that of Al–O under the same conditions; thus, the selectivity relative to side reactions is lower for lanthanide alkoxide in comparison to aluminum alkoxides, in which transfer reactions are almost nonexistent [69].

The first example with significant stereocontrol was reported by Le Borgne and Spassky [70]. They used simple alkylmetals (i.e., ZnEt_2_, CdMe_2_ and AlEt_3_) with (*R*)-3,3-dimethyl-1,2-butanediol for the polymerization of *rac*-BL. In the presence of ZnEt_2_ and AlEt_3_-based systems, the (*R*)-enantiomer of BL polymerizes; at low conversions, this leaves the unreacted (*S*)-enantiomer with an excess of 46% when using Zn. Fractionation of the crude polymer yielded a predominantly crystalline isotactic fraction and an amorphous heterotactic fraction, both of which were optically active. Interestingly, in the case of CdMe_2_, polymerization proceeded with much lower stereoselectivity, yielding a polymer that preferentially incorporated the (*S*)-enantiomer. The actual initiators that mediated all processes were chiral metal glycoxides.

### 4.2. Initiation with Discrete Heteroleptic Metal Complexes

**Scheme 39:**
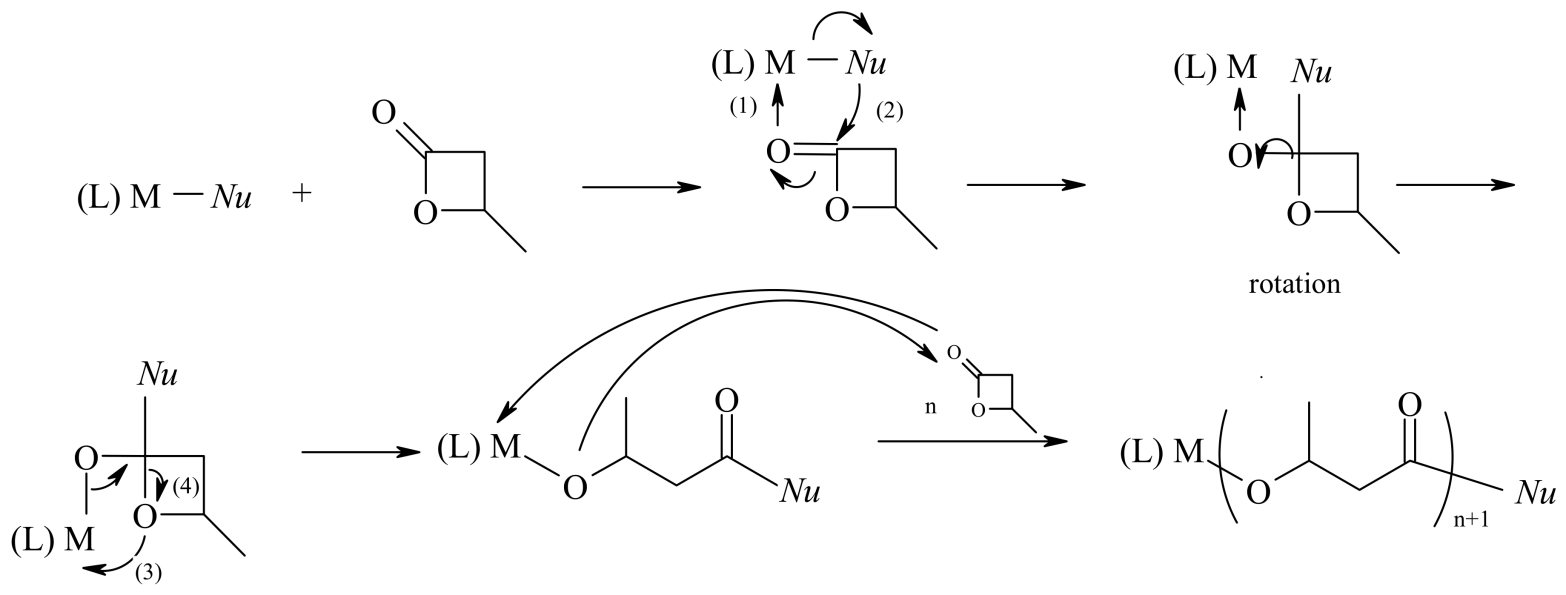
BL polymerization mediated with discrete organometallic complexes by a coordination–insertion mechanism.

Some metal-based initiators for the polymerization of β-substituted-β-lactones display exceptionally high activity. They are discrete heteroleptic organometallic complexes of the type (L)M–*Nu* (L: ancillary ligand; *Nu:* nucleophilic group as alkoxide, amide, etc.), relying on highly oxophilic metal centers, such as transition metals (Groups 3–12, block *d*)—e.g., Zn [71,72,73,74,75,76,77,78,79], Cr [74,75,76], Ti [77,78,79], and Zr [80,81,82,83]—as well as rare earth metals (Group 3, block *f*)—e.g., Y and Ln [84,85]. The coordination–insertion mechanism is typically used in metal-compound-initiated β-lactone polymerization [63] (Scheme 39).

The process begins with the coordination of the lactone to the unsaturated metal center (1), followed by nucleophilic attack of the active nucleophilic group on the carbonyl carbon of the lactone (2). It results in the insertion of the lactone into the M-*Nu* bond. After bond rotation, the migration of metal to the ethereal oxygen occurs (3), followed by rearrangement of the four-membered cyclic intermediate, resulting in the cleavage of the O-acyl bond (4) and the formation of metal–alkoxide species. The latter initiates further polymerization by the same mechanism.

**Scheme 40:**
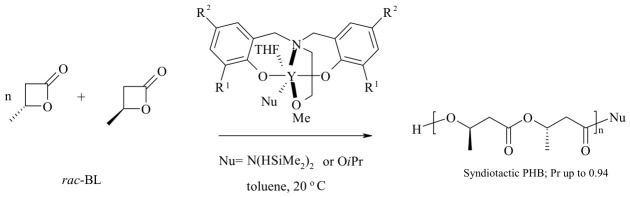
Living syndiospecific ROP of *rac*-BL promoted by amino-alkoxy-bis(phenolate) yttrium amido and alkoxide complexes [84].

One example is the diiminate zinc alkoxide complexes that polymerize *rac*-BL [86] with good rates and yields under mild conditions, giving atactic PBL. Another is the ROP of *rac*-BL initiated with chromium (III) salophen complexes at 100 °C, which allows the formation of isotactic-enriched PBL with high molar mass, yet with extremely broad dispersity [87]. However, the most syndiospecific class of catalyst for the ROP of *rac*-BL comprises discrete complexes of Group 3 and rare earth metals [85]. These catalysts display high activities and productivities under very mild conditions, as well as allowing for a significant degree of control over the polymerization in order to prepare polyesters with a controlled macromolecular architecture. With amino-alkoxy-bis(phenolate) yttrium amido and alkoxide complexes, the full conversion of 200–600 equiv. of *rac*-BL took place within 1–60 min at 20 °C in toluene solution (Scheme 40) [84]. The synthesized polymers had a high M_n_ and low M_w_/M_n_ = 1.05−1.15.

**Scheme 41:**
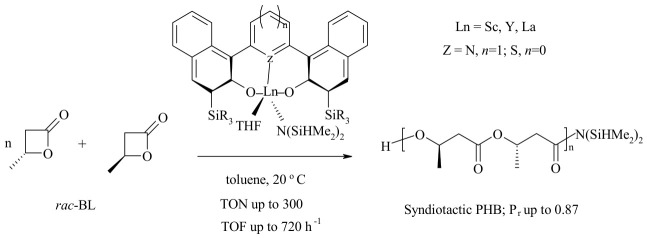
Syndiospecific ROP of *rac*-BL promoted by 2,6-bis(naphtholate) donor rare earth metal amino complexes [90].

NMR spectroscopy analysis confirmed that the ROP of *rac*-BL leads to a polyester chain selectively capped by CONR_2_ [NR_2_: N(SiHMe_2_)_2_] and COOR (OR: O-*i*-Pr), respectively. Polymerization occurs via a coordination–insertion pathway, with selective acyl–oxygen bond cleavage [84]. An interesting feature of these catalyst systems is the possibility to finely tune the selectivity via the nature of the R substituent on the phenolate ligand. High syndiotactity levels were observed with complexes having aryl-derived substituents (i.e., cumyl, trityl). This observation suggests that stereoselectivity cannot be explained by steric effects and that electronic influences may also need to be considered. Syndio-selectivity originates from pure chain-end control, which is well known to be assisted by the initiator due to the presence of sterically bulky ligands. Thus, it was supposed that other discrete rare earth metal complexes supported by different ligands would also enable the syndiospecific ROP of *rac*-BL [88,89]. One example is Sc, Y, and La complexes based on sterically demanding silyl *ortho*-substituted tridentate 2,6-bis(naphtholate) pyridine and 2,5-bis (naphtholate) thiophene ligands [90]. Such ligands can confer various symmetries at the metal center by the non-coplanar orientation of the rigid flat control heterocyclic donor and adjacent naphtholate groups, due to steric repulsion between these moieties (Scheme 41) [90].

**Scheme 42:**
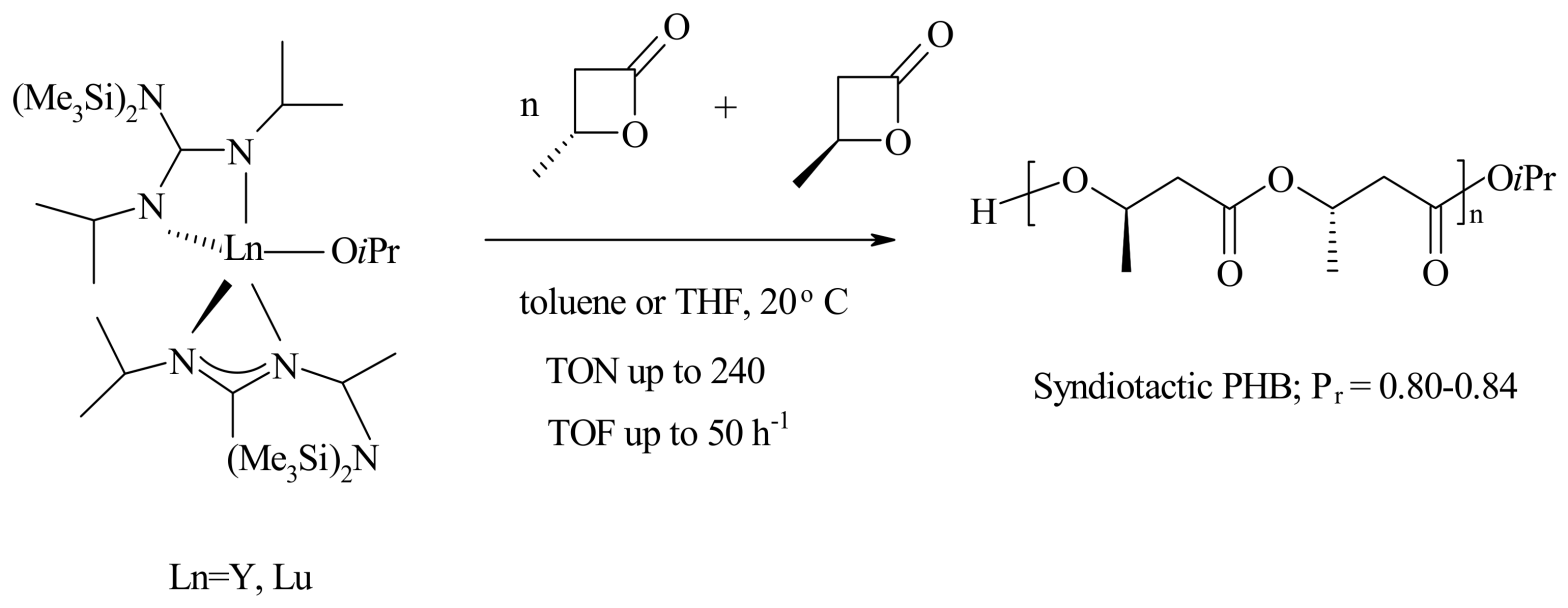
Bis(guanidinate) alkoxide group 3 metal complexes for ROP of *rac*-BL [88].

Moreover, a series of new bis(guanidinate) alkoxide metal complexes [(Me_3_Si)_2_NC-(N-*i*-Pr)_2_]_2_ LnOR (R: O-*t*-Bu, Ln: Y, Nd, Sm, Lu; R: O-*i*-Pr, Ln: Y, Nd, Lu) were used [88]. They afford PBL with relatively narrow dispersity and molar masses in good agreement with the calculated values. Bis(guanidinate) yttrium and lutetium isopropoxide complexes proved able to control the stereoselective ROP of *rac*-BL, yielding syndiotactic-enriched polyester (Scheme 42) [88].

Extensive research in recent years has demonstrated that diamino- or amino-alkoxy-bis(phenolate)yttrium amido catalyst systems are highly efficient for the stereoselective ROP of several β-lactones with various exocyclic side-groups in β-positions [91,92,93,94]. For example, these yttrium complexes successfully promote the highly stereoselective ROP of PL with R = All, Bn, CH_2_OMe, CH_2_OAll, or CH_2_OBn. The “non-covalent” interactions between the chemical nature of the exocyclic functional side-group on the β-lactone and the stereoelectronic tuning arising from the phenolate ligand substituents within the yttrium coordination sphere modulate the stereocontrol of ROP [94].

**Scheme 43:**
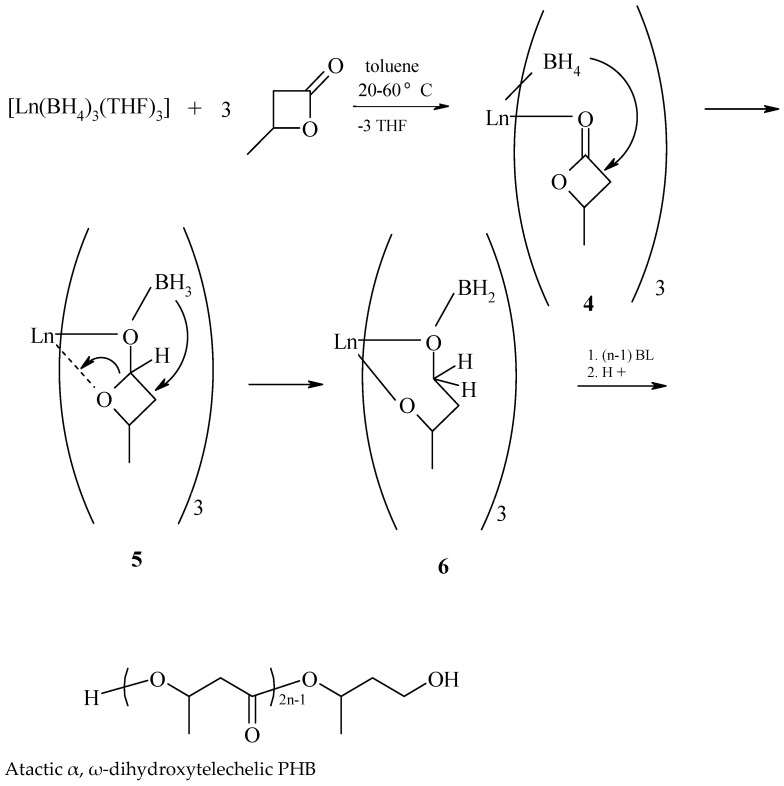
Polymerization of BL initiated by a rare earth metal trisborohydride complex [Ln(BH_4_)_3_(THF)_3_] [95].

In 2013, the simplest rare earth metal homoleptic trisborohydride complexes [Ln(BH_4_)_3_(THF)_3_] with Ln: La, Nd, and Sm were reported by Guillaume et al. [95] for the ROP of BL polymerization. Such complexes feature BH_4_^−^ ligands, in which hydrogen atoms bridge the metal center and the boron atoms. Based on the analytical evidence, the mechanism of the initiation step of polymerization involving two successive BH activations was proposed by the authors, as presented in Scheme 43 [95].

PHB-diols have been used as precursors to triblock polyesters or polyurethane copolymers. They were initially synthesized via the anionic ROP of BL using 4-hydroxybutanoic acid sodium salt/18C6 complex as initiators, followed by termination with bromo-alcohol. PHB-diols prepared through the use of triborohydride complexes in mild conditions are highly valuable, particularly as synthetic building blocks for tailor-made polymer materials [95].

The initiation first involves the displacement of THF upon coordination of BL to give [Ln(BH_4_)_3_(BL)_3_] **4**, followed by the hydride transfer to the oxygen atom to form **5** (first BH activation). Finally, ring-opening of the BL unit via acyl–oxygen bond cleavage with a second hydride transfer onto the same carbon affords **6**. Subsequent propagation goes on from this active rare-earth alkoxide species. Upon hydrolytic termination/deactivation, the ketone is reduced while the Ln–O bond is hydrolyzed, thus affording atactic poly(3-hydroxybutyrate) (PHB) (M_n_ up to 8000, M_w_/M_n_ ≤ 1.02).

The related isospecific *rac*-BL polymerization has been reported using silica-supported rare earth trisborohydride complexes SiO_2_[Ln(BH_4_)_3_(THF)_2_] [89]. The latter was shown to be more stereoselective than the unsupported catalysts.

Recently, Rieger et al. [96,97] reported the highly isoselective ROP of *rac*-BL using in situ generated catalysts, based on Y[N(SiHMe_2_)]_3_ (THF)_2_ as a precursor with the respective salan pro-ligand. These catalysts produced syndiotactic-enriched, isotactic-enriched, or atactic polymers, depending on the substitution pattern.

Furthermore, Jiang et al. [98] have studied the “living” ROP of BL initiated by mononuclear zirconium compounds containing sterically hindered *N, O*-chelate and anionic dimethylamide ligands at room temperature. The polymers obtained were biodegradable and had high M_n_ = 12,000 and narrow polydispersity. However, at 80 °C, *trans*-crotonate groups were formed, indicating β-proton elimination. This mechanism was predominant at elevated temperatures due to the detrimental effect of the generated H_2_O on the Zr initiator and the insufficient strength of the Zr-O-Zr species to initiate polymerization. The prepared polymers had an atactic configuration.

It is worth noting that some main-group metal (2 and 13) [99,100] complexes supported by a ligand have also been applied for the initiation of BL ring-opening polymerization. The results were similar to those obtained with transition metals or rare earth metals as initiators [99].

Young et al. [100] reported that (*S, S*)-prophenol Mg_2_(μ-O^n^Bu) (THF)_2_ is a highly enantioselective catalyst of *rac*-BL polymerization, giving isotactic PBL. This bimetallic magnesium catalyst polymerizes (*R*)-BL selectively with inversion of the configuration to (*S*)-PBL at a high rate (k_R_/k_S_ = 140).

The diphenoxylimine five-coordinated aluminum complex AlMeL_2_ (L = N-(2,6-diisopropylphenyl) phenoxyimine) is an active ROP catalyst supporting well-controlled living polymerization of *rac*-BL at 100 °C (with M_w_/M_w_ = 1.03) in the presence of BnOH, providing a biodegradable polyester. The suggested active species of this catalytic process is the aluminum complex Al(OBn)L_2_ [101].

The highly controlled living ROP of BL has been performed by using an ethoxy-bridged dinuclear indium catalyst [(NNO)InCl](μ−Cl)(μ−OEt) [102], which showed high activity and control during polymerization, allowing for the formation of diblock polyesters. The addition of a high amount of alcohol to the catalyst led to a fast chain transfer reaction. Moreover, an indium complex supported by the ferrocene-derived Schiff base ligand—i.e., (salfen) In(O*^t^*Bu)—had an unprecedented high activity toward BL [103], leading to extremely high-molar-mass polymers and low polydispersity in minutes. Similarly, other authors [104] have described an alkoxide indium complex with a salan-type structure, which had a high reaction rate, excellent control, and produced living polyesters with high molar masses.

Gaining knowledge on the roles of the group 13 metals, when combined with the appropriate ligand, in promoting a given mechanism would undoubtedly be beneficial for the discovery and development of potent stereoselective catalysts for controlled ROP of cyclic esters.

In summary, coordinated ROP is a very well-established and popular method for the synthesis of poly(hydroxyalkanoates). Discrete metal complexes are the only ones to effect significant stereocontrol in the ROP of chiral β-lactones. The catalyst plays a key role in the production of polyesters and determines their potential applications [105]. The fine-tuning of the organometallic catalyst, which initiates the chain-growth process and enchains all subsequent cyclic ester monomer units, allows for precise control of the molar mass, terminal groups, and the microstructure of the polymers [85,106]. Over the past two decades, much attention has been focused on the ROP of *rac*-BL leading to the synthesis of syndiotactic polyester (*s*-PBL). However, it is more difficult for BL to undergo stereoselective ROP for the synthesis of isotactic polyester (*i*-PBL) [107], which is generally produced via microbial fermentation [108], since there is currently no universal biodegradable polyester that can fulfill all requirements for a range of applications.

## 5. Supramolecular-Based ROP

**Scheme 44:**
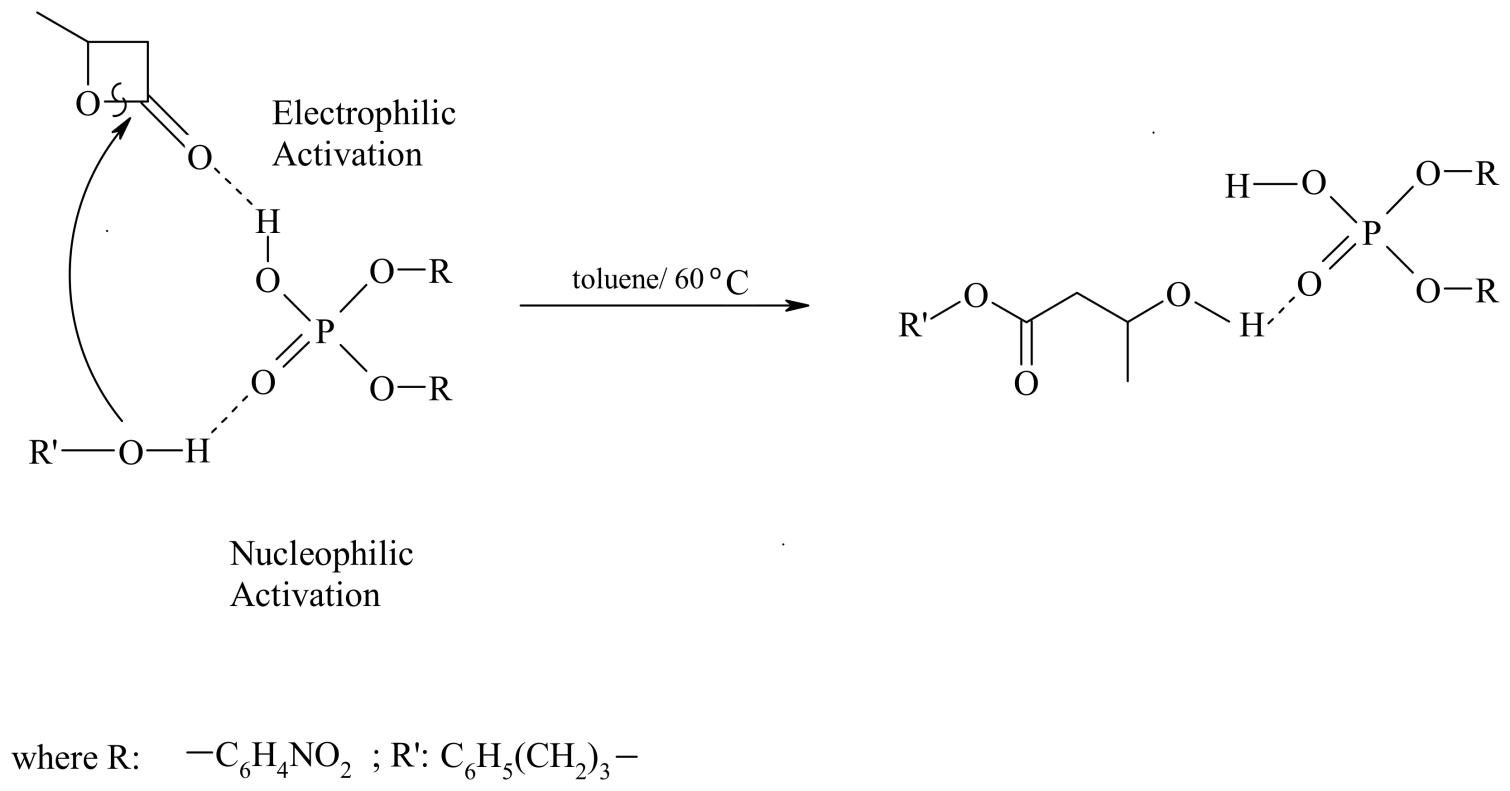
BL ROP mediated by a dual activation process from a BNPP catalyst [109].

In 2014, Kokuchi et al. [109] investigated BL polymerization mediated by 3-phenyl-1-propanol and diphenyl phosphate (DPP) or the more acidic bis-(4-nitrophenyl)phosphate (BNPP). Polymerizations were performed for [BL]_o_/[I]_o_ = 50 and [I]_o_/[BNPP]_o_ = 2, resulting in polyesters with DP_n_ = 20 ÷ 120. The authors proposed that the BNPP catalyst has a dual activation ability, as the hydroxyl proton of the phosphoric acid activates the monomer while the phosphoryl oxygen acts as a base, activating the initiating/propagating alcohol through the formation of hydrogen bonds (Scheme 44) [109]. The proposed mechanism does not involve ions.

A similar dual interaction effect through a “supramolecular interacting” system has been observed by Harada et al. [110,111] in BL polymerization mediated by α- and β-cyclodextrins (CDs). They both initiated and catalyzed polymerization in bulk at 100 °C by forming an inclusion complex with the monomer. A combination of CDs formed a hydrogen bond between the carbonyl oxygen of BL and the OH group of the CD to activate the monomer in the CD cavity.

**Scheme 45:**
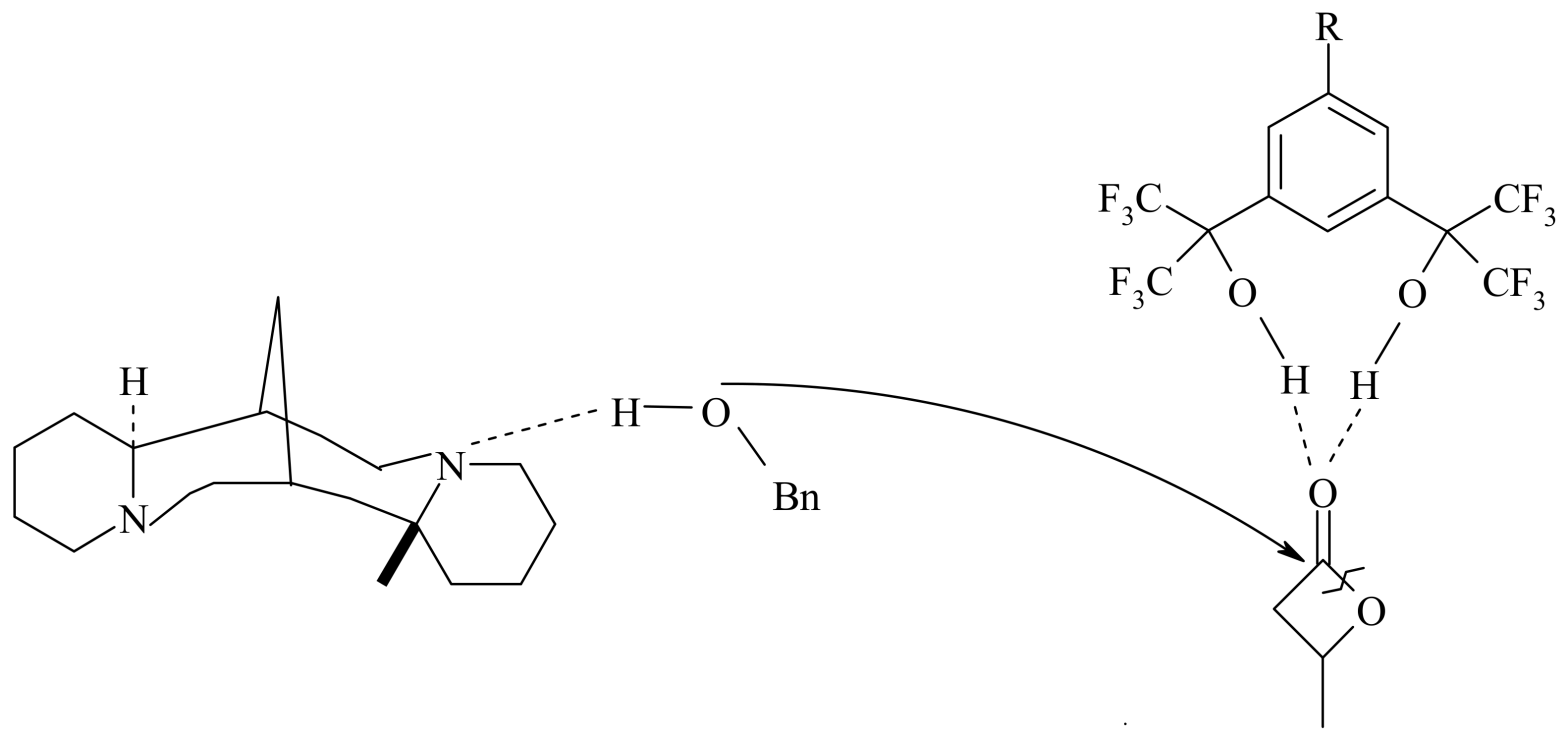
Bimolecular activating process implied in BL polymerization as promoted from benzyl alcohol using an HFA/SP catalytic complex (R: methacryloyl) [112].

Another interesting and efficient “multiple reversible non-covalent activating” system was used by Hedrick et al. [112]. The authors indicated that BL polymerizes in benzene with benzyl alcohol as the initiator, 3,5-bis-(1,1,1,3,3,3-hexafluoro-2-hydroxypropyl)phenyl methacrylate (HFA) as the catalyst, and (-)-sparteine (SP) as the co-catalyst (Scheme 45) [112].

Organic catalysis has proven to be of broad utility for the generation of poly(β-lactones). These methods have strong advantages over metal-based systems and allow for the development of new metal-free catalysts. This has permitted scientists to synthesize new lactones, such as α, α’β-transubstituted lactones and their corresponding polymers, which can be applied in the fields of nanomaterials and medicine.

## 6. Enzymatic ROP

Recently, the enzyme-catalyzed ROP of β-lactones has rapidly developed as a new approach for polyester synthesis, serving as an alternative to metal-based catalytic processes (biocatalysis) [113,114,115,116,117,118,119,120,121]. Novel lipases or esterases are used, derived from thermophiles, immobilized enzymes, and recombinant whole-cell biocatalysts. In enzymatic ROP, “green solvents” are applied, including water, ionic liquids, supercritical carbon dioxide, and hydrofluorocarbon solvents [119]. Enzymatic polymerization has many valuable features, including high catalytic activity, mild reaction conditions, high control of enantio-, chemo-, and regioselectivity, and few side products. The mechanism of lipase-catalyzed ROP of β-lactones is generally accepted to be a monomer-activated process [116]. Namekawa et al. [122] examined the polymerization of PL using various powdery lipases of different origins for the production of polyesters. PL was polymerized using *Pseudomonas* family lipases as catalysts in bulk, yielding a mixture of linear and cyclic oligomers with a molar mass of several hundred.

**Scheme 46:**
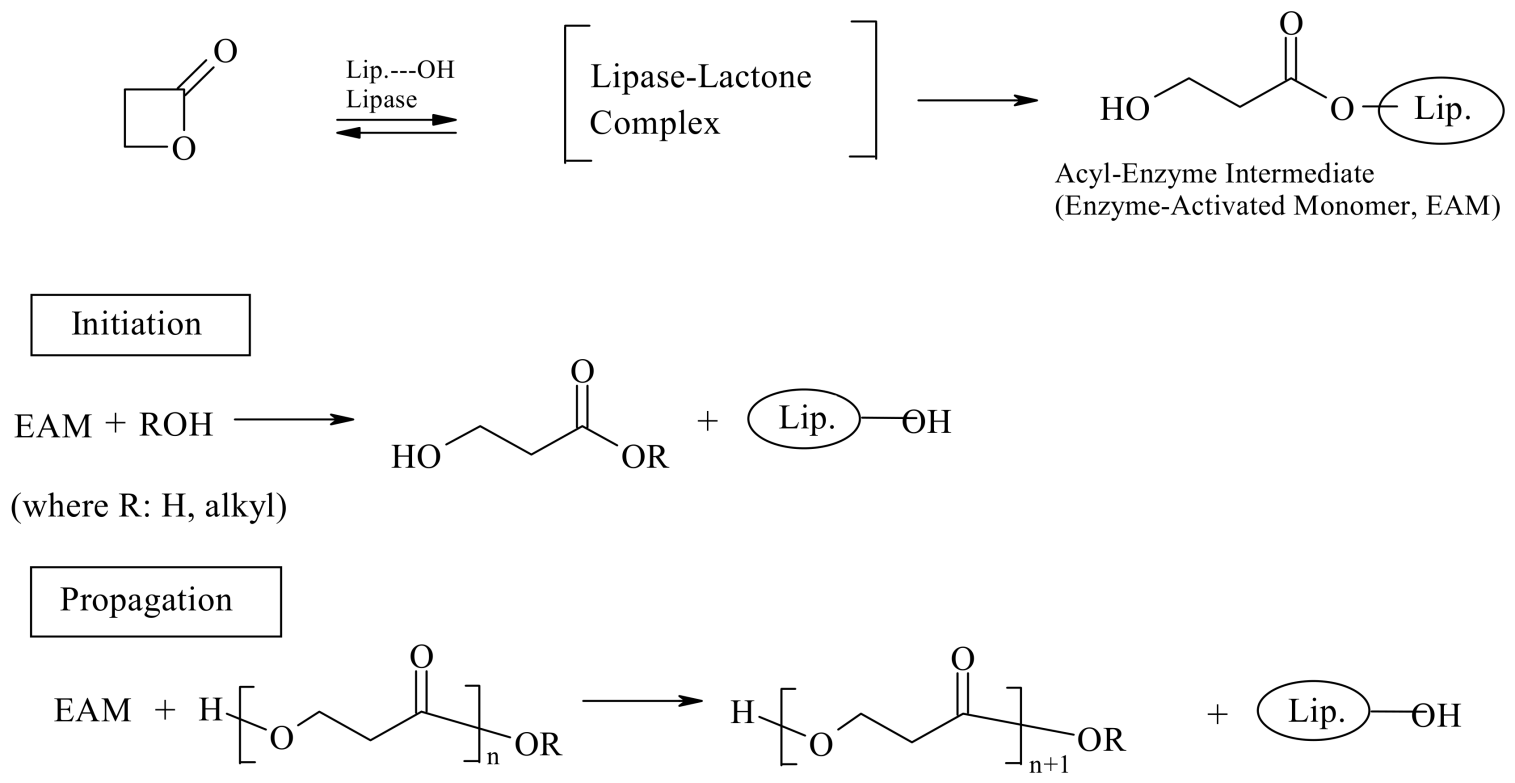
Postulated mechanism of enzymatic polymerization of unsubstituted lactone [116].

In 1999, Kobayashi et al. [116] proposed the mechanism of enzymatic polymerization of unsubstituted lactones with different ring sizes, including PL (Scheme 46).

Lipase-catalyzed reactions occur via a monomer-activated mechanism, which is nonionic in nature. The enzymatic polymerization of lactones is explained by considering the presented reactions as the principal reaction course. The catalytic site of lipase is known to be a serine residue. The key step is the reaction of the lactone with lipase, involving the ring-opening of the lactone to give the acyl–enzyme intermediate (enzyme-activated monomer, EAM). The initiation is a nucleophilic attack of water, which is probably contained in the enzyme, onto the acyl–carbon of the intermediate to produce ω-hydroxycarboxylic acid. In the propagation stage, the intermediate is nucleophilically attacked by the terminal hydroxyl group of a propagating polymer to produce a one-unit, more elongated polymer chain by O-acyl bond cleavage.

**Scheme 47:**
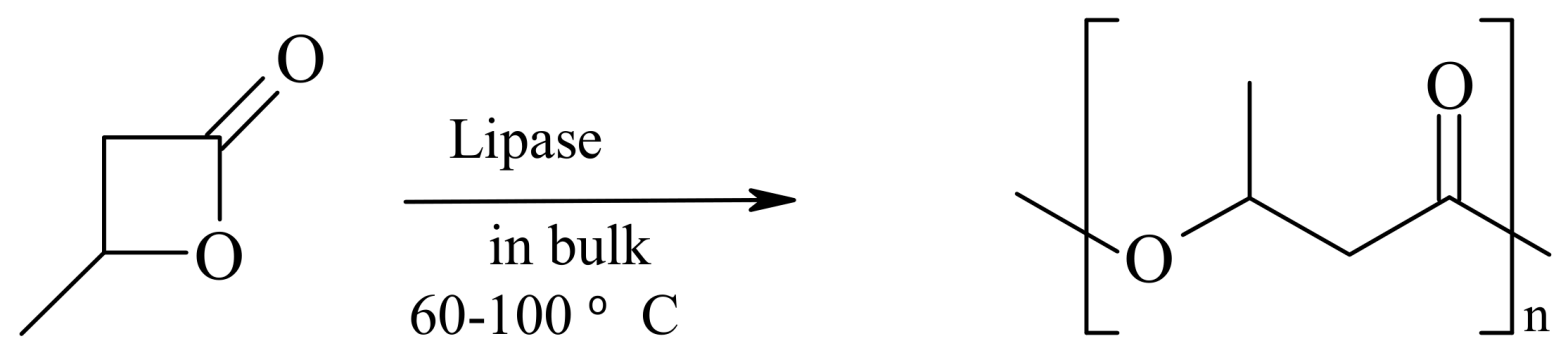
Enzymatic polymerization of BL resulting in a linear polyester via O-acyl bond cleavage [113].

Wang et al. [113] first examined the thermophilic lipase-catalyzed stereoselective ROP of racemic BL (Scheme 47).

According to the mechanism proposed for the lipase-catalyzed polymerization of other lactones, polymerization was initiated through reaction of the enzyme with the lactone to form an acyl–enzyme intermediate. The latter then reacts either with a nucleophile to accomplish the initiation or with the OH group of a growing polymer chain to continue the propagation. The formation of *R*-enriched optically active polymers can be attributed to the rate difference between the reaction of the lipase with (*R*)- or (*S*)-BL and/or the rate difference between the reaction of the acyl-enzyme intermediate with *R*- or *S*-configuration chain ends.

**Scheme 48:**
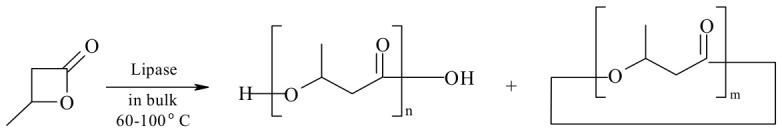
Lipase-catalyzed ROP of BL to the linear and cyclic polyesters [114].

Further studies on the enzymatic ROP of BL were continued by Matsumura et al. [114]. They tested two lipases, i.e., porcine pancreatis lipase and *Candida cyclindracea* lipase (at 80–100 °C). Both lipases showed the best results with respect to the molecular mass of the polymers M_n_(SEC) = 4000–7200 and the monomer conversion (92–99%). Aside from a linear polymer, a significant amount of the cyclic PBL fraction was produced, and the latter increased with monomer conversion (Scheme 48).

The authors observed that (*R*)-BL was more quickly polymerized compared to (*R,S*)-BL. The enzyme-catalyzed polymerization of BL may be affected by the polymer (oligomer) structures that EAM attacks. Isotactic and syndiotactic structures may vary the reactivity with the EAM. PBL consisting of (*R*)-BL units was more easily polymerized via the EAM reaction and reached a higher M_W_ than the corresponding (*R,S*)-PBL. Moreover, PBL produced via the ROP of (*R*)-BL had a linear fraction and cyclic ones. This phenomenon, as well as the broad molecular mass dispersion, might be ascribed to transesterification (intra- and intermolecular chain transfer reactions) [114].

In conclusion, the enzymatic ROP of β-lactones has been shown to be a powerful way to synthesize aliphatic polyesters under mild conditions. This method is environmentally friendly and could replace processes mediated by traditional toxic catalysts; it could also be used to produce biodegradable materials for medicine and drug delivery applications, providing a good example of “green polymer chemistry.”

## 7. Conclusions

**Scheme 49:**
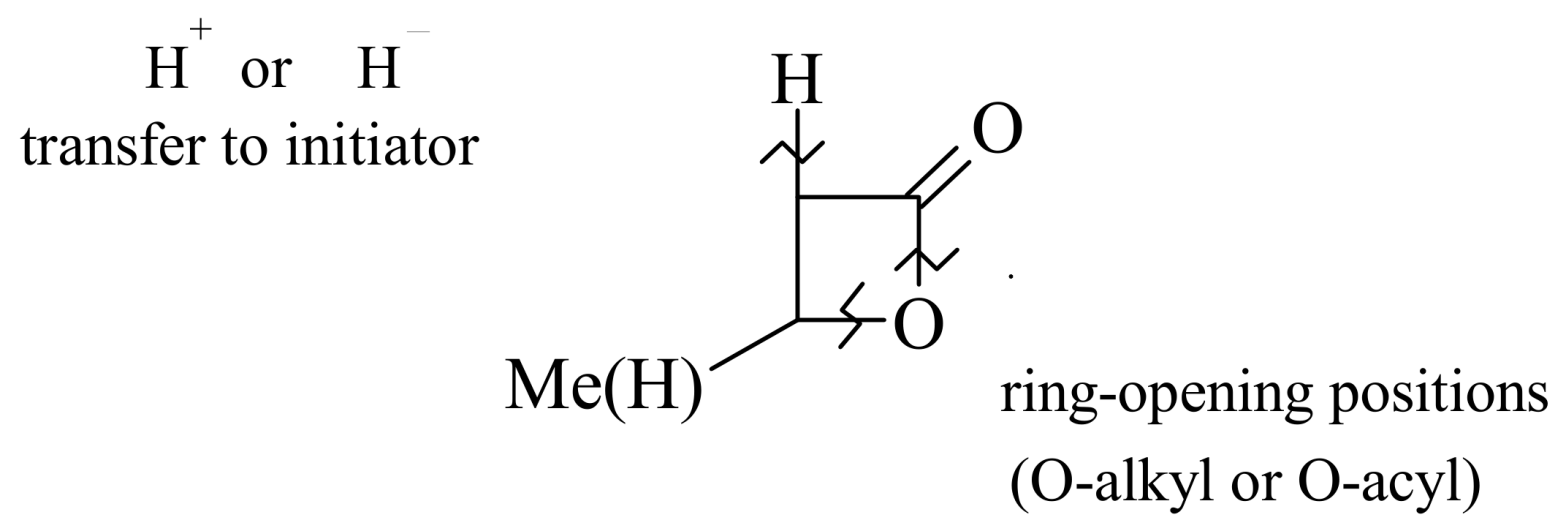
Bond cleavage during the initiation of β-lactone ROP.

The mechanisms of β-lactone polymerization depend strongly on the kind of initiator used. Several bond cleavage modes occur during the initiation step in the ring-opening polymerization (ROP) of PL. In general, one, two, or three bonds can be cleaved in a monomer molecule under the influence of some ionic or nonionic compounds, as shown in Scheme 49.

In the anionic ROP of BL, the C-H bond is exclusively cleaved under the influence of strong bases, such as K^+^H^−^, *t*-BuO-K^+^, or guanidine. After monomer deprotonation (proton transfer), the O-alkyl bond is cleaved, resulting in potassium *trans*-crotonate salt, which is the real initiator of polymerization.

Nucleophilic reagents, such as MeCOO-K^+^, MeCOO-(*n*-Bu)_4_N^+^, and monopotassium salts of dicarboxylic acids, initiate polymerization via direct nucleophilic attack of the anion on the C3 of the monomer, followed by ring-opening in the O-alkyl position.

Some initiators indicate dual behavior, also reacting as nucleophilic bases. For example, Ph_3_C^−^K^+^ reacts via monomer deprotonation as well as by O-alkyl bond cleavage, whereas MeO^−^K^+^ reacts via monomer deprotonation and O-acyl bond cleavage. Moreover, other nucleophilic bases, such as K^+^OH^−^ and PhO^−^Na^+^, react in three ways, i.e., monomer deprotonation and O-alkyl and O-acyl bond cleavage. However, in all anionic systems, propagation proceeds by O-alkyl bond cleavage.

Two-electron transfer reagents (e.g., K^−^K^+^L) initiate polymerization through the transfer of two electrons to the carbonyl group in two steps. The primary product is the β-lactone radical anion, following which the cyclic dianion is formed. The latter decomposes by ring-opening in the O-acyl position, giving K^+^OH^−^ and potassium crotonate.

The cationic polymerization of PL occurs via a different mechanism. The first step with carbenium cations of Me_2_Br^+^SbF_6_^−^ and acylum cations of MeCO^+^SbF_6_^−^ salts starts exclusively via an electrophilic attack of a cation on the exocyclic oxygen, which is much more nucleophilic than endocyclic ones, giving a cyclic tertiary oxonium cation. Propagation starts via nucleophilic attack of the exocyclic oxygen atom of the next monomer molecule on the β-carbon atom located at the chain end, then proceeds via O-alkyl bond cleavage (ACE mechanism). Interestingly, in polymerization mediated by tritylium cations of the Ph_3_C^+^SbCl_6_^−^ salt, nucleophilic transfer of the hydride anion H^−^ from the monomer (donor) to the electrophilic salt (acceptor) occurs. Then, the formed carbocation reacts with the next monomer molecule, giving an oxonium ion, and propagation proceeds according to the ACE mechanism.

In contrast, in the presence of the Ph_3_C^+^SbCl_6_^−^/ROH system, PL polymerizes via the hydroxyl mechanism. This consists of initiation by the intermediate oxonium salt RO^+^H_2_SbCl_6_^−^, which attacks the endocyclic carbon atom of the monomer. Propagation proceeds via the transfer of polymer to monomer via O-acyl bond cleavage.

Using an acid/alcohol system—e.g., CF_3_SO_2_OH (catalyst) and isopropanol (initiator)—for BL polymerization in the first step (activation), the proton from the acid attacks the exocyclic O-atom in the monomer. Then, the nucleophilic oxygen atom of the initiator attacks the C-atom of the carbonyl group (initiation), resulting in O-acyl bond cleavage. Propagation proceeds via an attack of the oxygen atom of the terminal hydroxy group on the carbon atom in the protonated monomer molecule (AM mechanism).

Many initiating systems have been applied for the coordinated ROP of BL. Various simple covalent metal alloxides can be used for this purpose, e.g., (*n*-Bu)_3_SnOMe, Al(O-*i*-Pr)_3_, Ti(O-*n*-Bu)_4_, Zn(O-*n*-Bu)_2_, and Zr(O-*n*-Pr)_4_. In this context, the nonionic coordination–insertion mechanism of initiation and propagation was proposed.

The first step is the complexation of the monomer at the carbonyl oxygen (the most basic and most nucleophilic site of the lactone) by covalent metal alkoxides. The next step is cleavage of the O-acyl bond of the lactone via the insertion of the lactone into the weakly polarized metal–oxygen bond. The proposed mechanism is characteristic of all metal alkoxides or phenoxides with free *p* or *d* orbitals, and results in the absence of trans-crotonate groups in the polymers obtained.

The second group of initiators is discrete organometallic complexes of the type (L)M-*Nu* (L: ancillary ligand, *Nu*: nucleophilic group as alkoxide or amide), relying on highly oxophilic centers of transition metals (M). Some of them give atactic PBL, and others provide isotactic-enriched PBL. The most syndiospecific class of catalysts for the ROP of BL comprises organometallic compounds based on rare earth metals (Group 3) with free *f* orbitals.

Metal-free supramolecular-based ROP initiated by non-covalent activated monomer/initiator systems—e.g., 3-phenyl-1-propanol (initiator) and bis-(4-nitrophenyl) phosphate (catalyst)—is quite different. The catalyst has a dual activation ability; in particular, the hydroxyl proton of the phosphoric acid activates the monomer while the phosphoryl oxygen acts as a base, activating the initiating/propagating alcohol via O-acyl bond cleavage.

Finally, enzymatic polymerization proceeds via a nonionic activated monomer mechanism, where the enzyme (lipase) reacts with β-lactone to give an acyl–enzyme intermediate. Next, the latter reacts with an alcohol used as the initiator. Then, polymerization proceeds via O-acyl bond cleavage.

Depending on the initiator used, the synthesized polyesters have different toxicities. Some toxicity may occur with polymers obtained with metal-based initiators (except Group 1 and 2 metals). These systems are applied in coordinated ROP (transition and rare earth metals, as well as Al, In, and Sn). In cationic ROP, the complex anions in ionic salts used as initiators may contain toxic metalloids, i.e., B, As, or Sb. Metal-free systems (organocatalysts and biocatalysts) are more environmentally friendly; however, β-lactones are classified as carcinogens [85]. Therefore, the prepared polyesters must be further purified in order to remove the residual unreacted monomer and metal contaminants, especially when polymers are designed for biomedical applications [53].

## Data Availability

No new data were created or analyzed in this study.
